# Large-Scale Benchmarking of Multireference Vertical-Excitation Calculations via Automated Active-Space Selection

**DOI:** 10.1021/acs.jctc.2c00630

**Published:** 2022-09-16

**Authors:** Daniel S. King, Matthew R. Hermes, Donald G. Truhlar, Laura Gagliardi

**Affiliations:** † Department of Chemistry, University of Chicago, Chicago Illinois 60637, United States; ‡ Department of Chemistry, Chemical Theory Center, and Minnesota Supercomputng Institute, University of Minnesota, Minneapolis Minnesota 55455-0431, United States; § Department of Chemistry, Pritzker School of Molecular Engineering, James Franck Institute, Chicago Center for Theoretical Chemistry, University of Chicago, Chicago Illinois 60637, United States

## Abstract

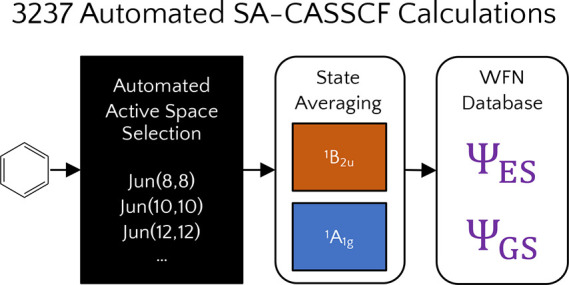

We have calculated state-averaged complete-active-space self-consistent-field (SA-CASSCF), multiconfiguration pair-density functional theory (MC-PDFT), hybrid MC-PDFT (HMC-PDFT), and *n*-electron valence state second-order perturbation theory (NEVPT2) excitation energies with the approximate pair coefficient (APC) automated active-space selection scheme for the QUESTDB benchmark database of 542 vertical excitation energies. We eliminated poor active spaces (20–40% of calculations) by applying a threshold to the SA-CASSCF absolute error. With the remaining calculations, we find that NEVPT2 performance is significantly impacted by the size of the basis set the wave functions are converged in, regardless of the quality of their description, which is a problem absent in MC-PDFT. Additionally, we find that HMC-PDFT is a significant improvement over MC-PDFT with the translated PBE (tPBE) density functional and that it performs about as well as NEVPT2 and second-order coupled cluster on a set of 373 excitations in the QUESTDB database. We optimized the percentage of SA-CASSCF energy to include in HMC-PDFT when using the tPBE on-top functional, and we find the 25% value used in tPBE0 to be optimal. This work is by far the largest benchmarking of MC-PDFT and HMC-PDFT to date, and the data produced in this work are useful as a validation of HMC-PDFT and of the APC active-space selection scheme. We have made all the wave functions produced in this work (orbitals and CI vectors) available to the public and encourage the community to utilize this data as a tool in the development of further multireference model chemistries.

## 1 Introduction

The accurate treatment of excited states is critical for understanding photochemical phenomena,^1−7^ and it has been a long-standing goal of the electronic structure community.^8−19^ Although treating excited states is difficult in general, it is particularly challenging when single-determinant methods such as Hartree–Fock or Kohn–Sham density functional theory provide a poor reference state for predicting excited states. This can occur either because the excited states vary greatly from the ground state (e.g., double excitations^20^) or because the ground state itself is not well described (e.g., strongly correlated systems^21−24^). One can overcome these deficiencies by using multiple-determinant reference states, and the methods that take this approach are called multireference methods.

The most popular multireference method is the complete active-space self-consistent field (CASSCF) method,^25^ which expresses approximate wave functions in the space of all possible configurations of electrons in an “active space” of orbitals and electrons. These wave functions can then serve as references for perturbation theories such as MC-QDPT,^26,27^ CASPT2,^28,29^ and NEVPT2.^30,31^ Alternatively, quantitative accuracy can be achieved by using a nonclassical-energy functional applied to the converged wave function in multiconfiguration nonclassical functional theory (MC-NCFT).^32−36^ The total energy is then a sum of the classical portion of the CASSCF energy and the nonclassical energy from the functional.

The most common form of MC-NCFT utilizes nonclassical-energy functionals obtained by translating Kohn–Sham exchange–correlation functionals for use with multiconfigurational wave functions via the on-top pair density and is called multiconfiguration pair-density functional theory (MC-PDFT). The translated PBE functional (tPBE) has been used as the functional in the majority of MC-PDFT calculations to date. The nonclassical energy from a density functional can be mixed with the nonclassical part of the CASSCF energy to form a “hybrid” nonclassical functional; for example, using a 0.75:0.25 mixture of tPBE and CASSCF nonclassical energies yields the tPBE0 functional.^37^

Difficulties encountered in all such post-CASSCF methods are making the active space large enough and well balanced enough to converge to a qualitatively accurate description of the underlying wave function(s). The results can depend significantly on the size and nature of the active space and the initial orbital guess.^38^ Moreover, in many occasions, the orbitals will change character during their optimization. For these reasons, such calculations often require expert human guidance to carefully choose and monitor the active space size and composition.

Although CASSCF has been used since the 1980s,^25^ the prospect of automated active space selection has only received significant attention within the last decade or so.^39−58^ Recently, we published the ranked-orbital approach to select active spaces and the approximate pair coefficient (APC) approximation for low-cost estimates of the orbital entropies used in the ranking.^59^ This automated scheme, inspired by the entropy-driven approach of Stein and Reiher,^42^ allows for the flexible selection of active-space size with a hierarchy of levels (max(8,8), max(10,10), max(12,12)...) reminiscent of the CI level sequence (CISD, CISDT, CISDTQ, ...).

Recently, Jacquemin and co-workers published the QUESTDB benchmark data set of 542 vertical excitation energies on a diverse set of small and midsize main-group molecules, calculated via a variety of high-level wave function methods in the aug-cc-pVTZ^60,61^ basis.^20,62−66^ In the present paper, we have undertaken the automated calculation of these excitation energies with SA-CASSCF, NEVPT2, and MC-PDFT using the APC-ranked-orbital active-space selection scheme. To benchmark and analyze the performance of various multireference methods on this diverse set of excitations, we eliminate poor active spaces (20–40% of calculations) by setting an error threshold on the SA-CASSCF excitation energy because that has previously been shown to be good way to judge the quality of the active space.^49^

By analyzing results across different active space and basis set size choices, we find different trends in the performance of MC-PDFT and NEVPT2 where the performance of NEVPT2 is overly dependent on the basis set in which the underlying wave function is converged. Additionally, we are able to produce the first large-scale and robust comparison of MC-PDFT to other single-reference methods such as CC2 and find the CASSCF mixing parameter of 0.25 used in tPBE0 to be optimal. We have made all the wave functions converged in this work available to the public via publication of the converged orbitals and CI vectors and encourage others to use these data in the development of further multireference model chemistries.

## 2 Methods

### 2.1 Data Overview

The data we have examined can be found in the QUESTDB data set,^65^ which consists of 542 vertical excitations of small and midsize main-group molecules (molecules with 1–10 non-hydrogenic atoms). Of these excitations, 491 are from singlet ground states and 51 are from doublet ground states. Every excitation in the QUESTDB data set is specified by its spatial and spin symmetries, and benchmark values are reported as “theoretical best estimates” (TBEs) calculated with a variety of high-level methods with the aug-cc-pVTZ^60,61^ basis. These TBEs have been used in this work to judge the errors of all computed excitation energies, even those obtained with a different basis set.

### 2.2 Active-Space Selection

To obtain orbitals for the active-space selection scheme, we started with a restricted Hartree–Fock singlet wave function for closed-shell molecules and a restricted open-shell Hartree–Fock doublet wave function for doublet molecules, as calculated using PySCF.^67^ The molecular point group was reduced to the highest available symmetry implemented for the PySCF SA-CASSCF solver: *C*_2*h*_, *C*_2*v*_, *C*_*s*_, or *D*_2*h*_. The APC-ranked-orbital active-space selection scheme^36,59^ starts with a set of candidate localized orbitals, ranks them by their approximated orbital entropies, and then eliminates orbitals starting from the lowest-entropy orbitals (those with the highest entropies are considered to be the most important) until the active space size reaches a predetermined maximum number of configuration state functions. We next describe the generation of candidate orbitals, then the ranking scheme, and finally the maximum-size criteria.

Following previous work,^36^ up to 23 lowest-energy virtual orbitals of the Hartree–Fock calculation were selected, and orbitals within this subset were grouped by symmetry and Boys-localized^68^ within each symmetry. Likewise, up to 23 highest-energy doubly occupied orbitals were also grouped by symmetry and Boys-localized within each symmetry. These two sets of localized orbitals (and the one singly occupied orbital, when present) were then considered as candidates for the active space. Next, we describe how we ranked the localized orbitals.

In the originally published APC ranking scheme, given a set of doubly occupied candidate orbitals *i* and virtual orbitals *a*, the APC matrix C_*ia*_ was calculated as
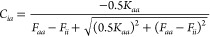
1where *K*_*aa*_ is the diagonal virtual element of the exchange matrix and *F*_*aa*_ and *F*_*ii*_ are diagonal virtual and doubly occupied elements of the Fock matrix in the MO basis. The entropies of doubly occupied orbitals *i* are then calculated as
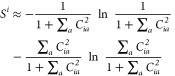
2and those of virtual orbitals *a* as
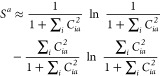
3

Finally, any singly occupied orbitals are assigned the maximum entropy value from the set of doubly occupied and virtual orbital entropies ({*S*_*i*_}, {*S*_*a*_}). Note that the removal of a virtual orbital from consideration affects all doubly occupied entropies and vice-versa. This method is inexpensive because it uses only easily calculated diagonal Hartree–Fock matrix elements (Supporting Information Section S1.3).

However, in the present work, we have found that in larger molecules (with >350 aug-cc-pVTZ basis functions), the APC entropies tend to overestimate the interaction of some virtual orbitals with the doubly occupied orbitals, artificially inflating the entropies of all doubly occupied orbitals and causing the selection of highly imbalanced active spaces (Supporting Information Figure S1). To overcome this issue, we propose an algorithmic extension of APC in which high-entropy virtual orbitals are removed from consideration when calculating entropies and then assigned the maximum entropy value (i.e., treated in the same way as singly occupied orbitals). The algorithm takes the following steps:

- Provide the sets of candidate doubly occupied/singly occupied/virtual orbitals ({*L*_*i*_}, {*L*_*s*_}, {*L*_*a*_}).
- Calculate entropies ({*S*_*i*_}, {*S*_*s*_}, {*S*_*a*_}) = APC({*L*_*i*_}, {*L*_*s*_}, {*L*_*a*_}) and then remove the highest-entropy virtual orbital from *L*_*a*_ and put it in *L*_*s*_. Repeat *N* times.
- Return ({*S*_*i*_}, {*S*_*s*_}, {*S*_*a*_}).

The algorithm above has a single parameter *N* (APC-*N*), which is the number of times the highest-entropy virtual orbital is removed. In this work, we have found a good value of *N* to be 2 (a scheme we refer to as APC-2), and we find that using APC-2 entropies results in more balanced active spaces and lower SA-CASSCF error than APC (Supporting Information Figure S2). The APC-2 entropies are then used to rank and select the orbitals for the active space by dropping the lowest-entropy orbitals until the active space size is lower than some maximum active space size. However, we note that the current scheme should be improved for the treatment of orbitals with degenerate entropies (Supporting Information Section S6.4).

In more detail, the active space size for a given set of *N*_orb_ active orbitals containing *N*_elec_ active electrons is calculated via the equation^69^
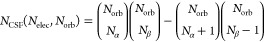
4where *N*_α_ + *N*_β_ = *N*_elec_ and *N*_α_ = *N*_β_ for even *N*_elec_ and *N*_α_ = *N*_β_ + 1 for odd *N*_elec_. The maximum active space size *N*_CSF_^Max^ is set via a specification of a maximum number of active electrons and orbitals (*N*_elec_^Max^, *N*_orb_^Max^) whose size is calculated via eq 4; this maximum active-space choice is notated as max(*N*_elec_^Max^, *N*_orb_^Max^). In this work, we calculate results at three choices of max(*N*_elec_^Max^, *N*_orb_^Max^): max(8,8) (*N*_CSF_^Max^ = 1764), max(10,10) (*N*_CSF_^Max^ = 19404), and max(12,12) (*N*_CSF_^Max^ = 226512). Following this specification, all orbitals are selected and then the lowest-entropy orbital is successively dropped until the size of the active space calculated via eq 4 is less than or equal to *N*_CSF_^*Max*^.

As an example, we guide the reader through choosing a max(4,4) active space (*N*_CSF_^Max^ = 20) from a set of orbitals with occupancies and entropies {(*n*_*j*_, *S*_*j*_)}
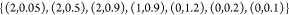
5

In this case, the active space is selected as follows:

- (2,0.05),(2,0.5),(2,0.9),(1,0.9),(0,1.2),(0,0.2),(0,0.1) | (7,7) *N*_CSF_ = 784
- (2,0.5),(2,0.9),(1,0.9),(0,1.2),(0,0.2),(0,0.1) | (5,6) *N*_CSF_ = 210
- (2,0.5),(2,0.9),(1,0.9),(0,1.2),(0,0.2) | (5,5) *N*_CSF_ = 75
- (2,0.5),(2,0.9),(1,0.9),(0,1.2) | (5,4) *N*_CSF_ = 20

with a resulting selected (5,4) active space.

### 2.3 Calculation of the Excitation Energies

Calculations of excited-state wave functions were carried out by state-averaged CASSCF, averaging over the ground state and the minimum necessary number of excited states of the symmetry specified by QUESTDB. For example, in a *C*_2*v*_ molecule (e.g., water), if the symmetry of the excited state under consideration is specified to be ^1^A_2_ with no lower ^1^A_2_ excitations present, then the state averaging was done evenly over the ^1^A_1_ ground state and the ^1^A_2_ excited state. For higher ^1^A_2_ excitations, however, the state averaging included an additional ^1^A_2_ state for each ^1^A_2_ excitation lower in energy (again with weights for state averaging being the same for all states averaged). Standard convergence parameters were employed, and for a few poor active-space choices (0.4% of cases), the calculations failed to converge.

Because the highest available point groups supported by the SA-CASSCF solver in PySCF have lower symmetry than those specified by QUESTDB for single atoms and diatomics, the point groups sometimes had to be reduced to the highest-symmetry subgroup. Additionally, the labeling of different irreps is sometimes a choice of axis convention, such as between B_1_ and B_2_ in *C*_2*v*_ or between B_1g_/B_2g_/B_3g_ and B_1u_/B_2u_/B_3u_ in *D*_2*h*_; we have done our best to match the irrep we think was used in QUESTDB. Calculations were done with the highest M_*S*_ allowed by the spin symmetry (e.g., if an excited state has *S* = 1, then for 8 electrons in the active space the active space would have 5 α and 3 β electrons).

The tPBE and NEVPT2 energies of the converged SA-CASSCF states were then calculated using the implementations of these methods in PySCF. Our implementation of MC-PDFT within PySCF is currently available in the mrh repository.^70^ Additionally, tPBE0 energies were calculated by averaging the SA-CASSCF and tPBE energies^37^
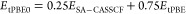
6

The only implementation of NEVPT2 currently in PySCF is strongly contracted NEVPT2 (SC-NEVPT2),^31^ and our NEVPT2 calculations use this.

In order to maintain a consistent labeling, the excited state to be compared to the QUESTDB excitation energy was chosen to be the state highest in energy as judged by tPBE. Although this is not a fail-proof scheme in terms of isolating the “same” QUESTDB state of the specified symmetry due to root flipping, we have found it to be satisfactory for our work as the converged QUESTDB wave functions are unavailable and labels such as “*n* → π*” are ambiguous non-observables. However, because the present work shows that tPBE0 is more accurate than tPBE, we suggest ordering the states by tPBE0 in future work.

### 2.4 Method Timing

All converged CASSCF wave functions (orbitals and CI vectors) were saved to disk at the end of the calculation. Timings for tPBE and NEVPT2 calculations were achieved by loading in the converged CASSCF wave functions, computing the relevant quantity (the tPBE nonclassical energy or the NEVPT2 perturbative correction) and then saving the results. The amount of resources requested for each calculation was determined by an empirically derived formula dependent on the number of aug-cc-pVTZ basis functions in the underlying molecule (Supporting Information Section S8.2), and so, timings between the tPBE and NEVPT2 implementations available in PySCF can be fairly compared (although we note that methodologies can always be further optimized).

### 2.5 Plotting

Figures were made in Python using matplotlib as enhanced by Pandas^71,72^ and Seaborn.^73^ Seaborn calculates 95% confidence intervals for the mean values reported in plots by bootstrapping the mean value over 1000 random samplings of the underlying data.^74−76^

## 3 Results

### 3.1 Eliminating Poor Active Spaces

We calculated excitation energies for all 542 vertical excitations listed in the QUESTDB database with six combinations of active space and basis set: four involving max(12,12) APC-2 active spaces with decreasing basis size (aug-cc-pVTZ,^60,61^ jun-cc-pVTZ,^77^ cc-pVTZ,^78,79^ and cc-pVDZ^78,79^) and two involving jun-cc-pVTZ with decreasing active space size [max(10,10) and max(8,8)]. We will refer to these combinations throughout the paper as Aug(12,12), Jun(12,12), TZ(12,12), DZ(12,12), Jun(10,10), and Jun(8,8).

**Figure 1:**
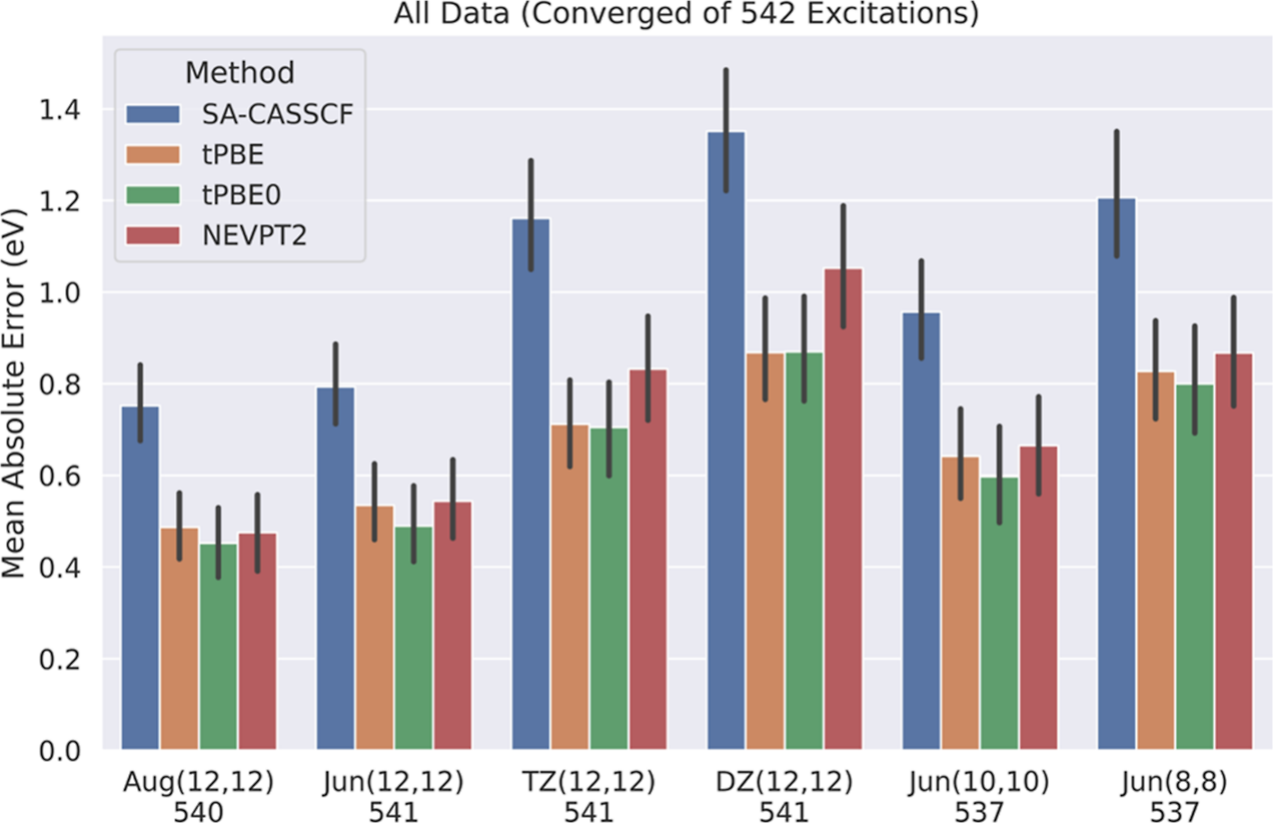
Comparison of the mean absolute errors of SA-CASSCF, tPBE, tPBE0, and NEVPT2 across different active space and basis set sizes for all converged calculations. The number of converged excitations with each combination of active space and basis is shown below each column, and 95% confidence intervals for each mean are shown in black.

Figure 1 shows the mean absolute errors of SA-CASSCF, tPBE, tPBE0, and NEVPT2 that we obtain for all wave functions converged at each combination of active space and basis set tested in this work. Adding together the number of converged calculations at each active space and basis set shown at the bottom of Figure 1 yields 3237 calculations. As expected, we find that the SA-CASSCF error increases when we move from a larger to a smaller basis set with a given active-space scheme or when we move from a larger active space to a smaller one with a given basis set. However, in order to reasonably evaluate the accuracies of these methods, we need to eliminate results whose error is driven mainly by poorly chosen active spaces. To analyze only cases with reasonable active spaces, we set a threshold *T* on the SA-CASSCF error of 1.1 eV (*T*_SA-CASSCF_ = 1.1 eV). That is, we consider that the APC scheme has produced a good active space if the error in the SA-CASSCF excitation energy is less than 1.1 eV.

**Figure 2:**
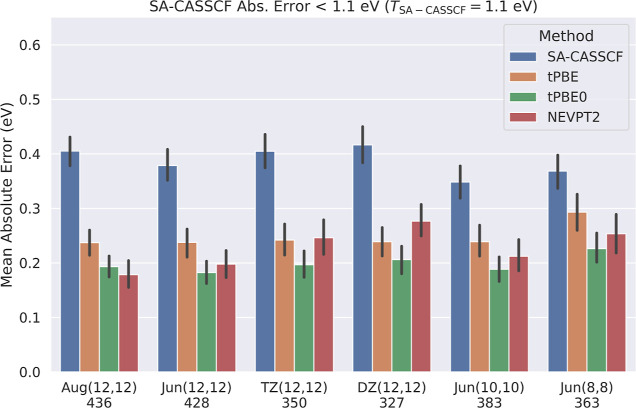
Comparison of the mean absolute errors of SA-CASSCF, tPBE, tPBE0, and NEVPT2 excitations across different active space and basis set sizes included by *T*_SA-CASSCF_ = 1.1 eV. The number of excitations included in this analysis for each combination of active space and basis set is shown below each group of bars, and 95% confidence intervals for each mean are shown in black.

Figure 2 shows the performance of SA-CASSCF, tPBE, tPBE0, and NEVPT2 at different active space and basis set sizes after using the 1.1 eV SA-CASSCF error cutoff to eliminate poor active-space choices. As expected, instead of observing an increasingly poor performance for SA-CASSCF excitations as active space and basis set size is decreased, we instead see a consistent error of roughly 0.39 ± 0.03 eV with an increasing amount of excitations excluded by *T*_SA-CASSCF_ = 1.1 eV. The number of excluded excitations roughly doubles from 19.2% at Aug(12,12) to 39.6% at DZ(12,12) with decreasing basis size and to 32.4% at Jun(8,8) with decreasing active space size. We note the very small increase of eight excluded excitations upon moving from Jun(12,12) to Aug(12,12), highlighting the very efficient nature of the jun basis set.^77^ Of course, with a better automatic active-space selection scheme, one would observe an increased amount of excitations included at each active space and basis set size, but the error will remain fairly consistent.

As we found for SA-CASSCF, we find that tPBE (0.25 ± 0.02 eV) and tPBE0 (0.20 ± 0.02 eV) maintain relatively consistent errors across different active spaces and basis set sizes when the 1.1 eV SA-CASSCF error threshold is applied. This is an intuitive result, as the accuracy of MC-PDFT is primarily contingent on the quality of the SA-CASSCF density and on-top density and on the quality of the on-top functional; if one eliminates the poor active spaces, then the functional (correlation) error may dominate, and this is approximately independent of the active space and basis set. In contrast, NEVPT2 shows quantitatively worse results as the basis set is decreased even as the wave function remains qualitatively well described. This makes sense because the power of NEVPT2 to change the SA-CASSCF energy stems from its perturber states, which are less capable of describing dynamic correlation within a smaller basis because the smaller basis set cannot represent the virtual-orbital space as well.^80−82^

**Figure 3:**
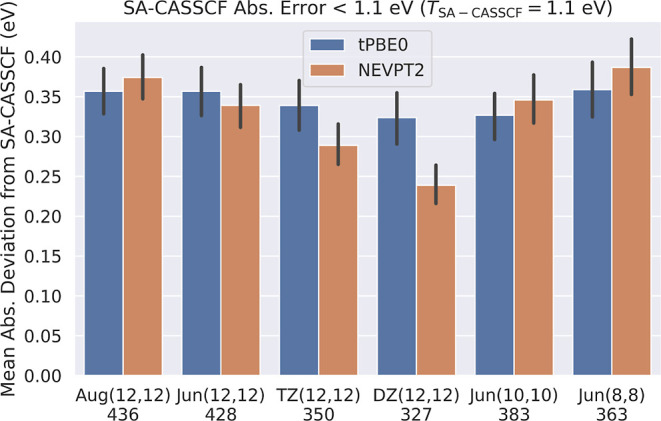
Mean absolute changes to the SA-CASSCF excitation energy made by tPBE0 and NEVPT2 across different active space and basis set calculations included by *T*_SA-CASSCF_ = 1.1 eV.

Figure 3 shows that the difference between the tPBE0 excitation energy and the SA-CASSCF excitation energy remains fairly consistent across active spaces and basis sets, but there is a significant drop in the NEVPT2 correction when moving from aug-cc-pVTZ to cc-pVDZ, resulting in increased NEVPT2 error. Figure 3 combined with Figure 2 shows clearly how the NEVPT2 results degrade in quality with decreasing size of the basis set, while the performance of tPBE0 remains consistent. As the basis set is decreased in size, the mean absolute change to the SA-CASSCF excitation energy decreases for NEVPT2, while remaining constant for tPBE. These results provide a plausible explanation of the discrepancy in mean absolute error found for SC-NEVPT2 between the study of Schapiro et al.^83^ (0.23 eV) and the more recent study of Sarkar et al.^84^ (0.15 eV). They imply that it is due to the fact that the Sarkar study used the aug-cc-pVTZ basis, while the Schapiro study employed the cc-pVTZ basis. However, our results point to this being caused by a poorer performance of NEVPT2 with the smaller basis set and not due to a poorer zeroth-order description of the underlying wave functions.

Further discussion of the error threshold is given in the Supporting Information, which shows the 1.1 eV SA-CASSCF error threshold to be optimal (albeit imperfect) for isolating subsets of automated wave function calculations that reproduce results curated by hand.^84^ Additionally, we analyze alternative error thresholds on the NEVPT2 and tPBE0 error. However, an error criterion cannot be used when a benchmark excitation energy or experimental excitation energy is not available. Nevertheless, when an accurate value is not available, one can still use this criterion (although with somewhat less reliably) by comparing to one’s best estimate rather than to an accurate value. Clearly, if one’s best estimate is good, this will work as well as comparing to an accurate value.

Finally, one might wonder what one can do to fix the active space if a calculation goes poorly. Of course, increasing the size of the active space via *N*_CSF_^Max^ is a worthwhile option to explore if affordable, and it is clearly seen in Figure 2 how this significantly increases the success rate of the selection algorithm. However, following our previous work,^59^ we also recommend experimenting with different orbital localization schemes for initializing the ranked-orbital selection as this can be a low-cost way to converge to a reasonable result.

### 3.2 Comparison to Single-Reference Methods

#### 3.2.1 Data Overview

In the QUESTDB database,^65^ excitations from many methods are only reported for the 491 excitations from closed-shell (*S*_0_) molecules, and, due to double excitations and strongly mixed states, results from most methods are only available for about 460 of these excitations (Supporting Information Figure S15). Our Aug(12,12) results comprise 436 excitations included by *T*_SA-CASSCF_ = 1.1 eV, 399 of which come from closed-shell molecules. Combining all methods and leaving out STEOM-CCSD, CCSDR(3), and CCSDT-3 for which there is significantly less available data (Supporting Information Figure S15), there are a total of 373 excitations consistently available for comparison with SA-CASSCF, tPBE0, NEVPT2, and 12 other methods in the QUESTDB database. Unlike Jacquemin and co-workers, we have not limited ourselves to comparisons based on “safe”^65,66^ excitations, and this includes 29 excitations that would otherwise have been excluded (out of a total of 57 unsafe excitations in the total set of 542).

**Figure 4:**
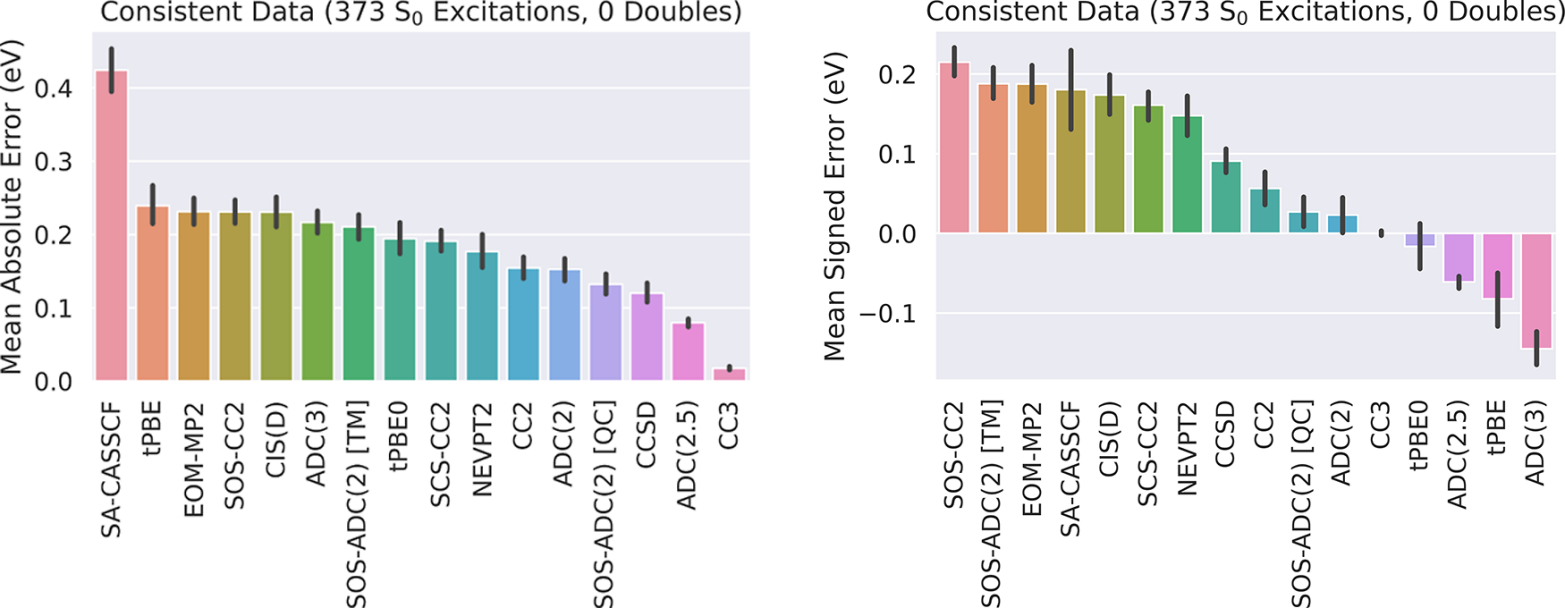
Comparison of the mean signed and unsigned errors of various methods on the 373 Aug(12,12) excitations included by *T*_SA-CASSCF_ = 1.1 eV error threshold. The 95% confidence intervals are shown in black. Left: Mean absolute errors. Right: Mean signed errors.

Figure 4 shows the mean absolute and signed errors of SA-CASSCF, tPBE, tPBE0, and NEVPT2 in comparison with 12 other methods in the QUESTDB database on the set of 373 excitations. First considering the mean absolute errors, we find that both NEVPT2 (0.18 eV) and tPBE0 (0.19 eV) have accuracy on par with CC2 (0.15 eV), with tPBE lagging significantly behind (0.24 eV). However, we note that the errors we report here for tPBE, tPBE0, and NEVPT2 are likely slightly overestimated, as our CASSCF error threshold is imperfect and fails to eliminate all cases with poor active-space choices (as discussed in the Supporting Information). Furthermore, this consistently available data set excludes all double excitations, for which the performance of the multireference methods is far superior (as discussed below).

Nevertheless, trends in the signed errors are particularly interesting, with all but four methods (ADC(3), ADC(2.5), tPBE, and tPBE0) overestimating excitations; this implies biased relative overstabilization of the ground state for most of the methods. One can clearly see how tPBE0 benefits from balancing the treatment of exchange and correlation, with SA-CASSCF overestimating excitations by 0.18 eV and tPBE underestimating by 0.08 eV, such that tPBE0 has nearly zero mean signed error. We note that the same good balance seems to occur in ADC(2.5),^85^ which averages ADC(3) and ADC(2).

**Figure 5:**
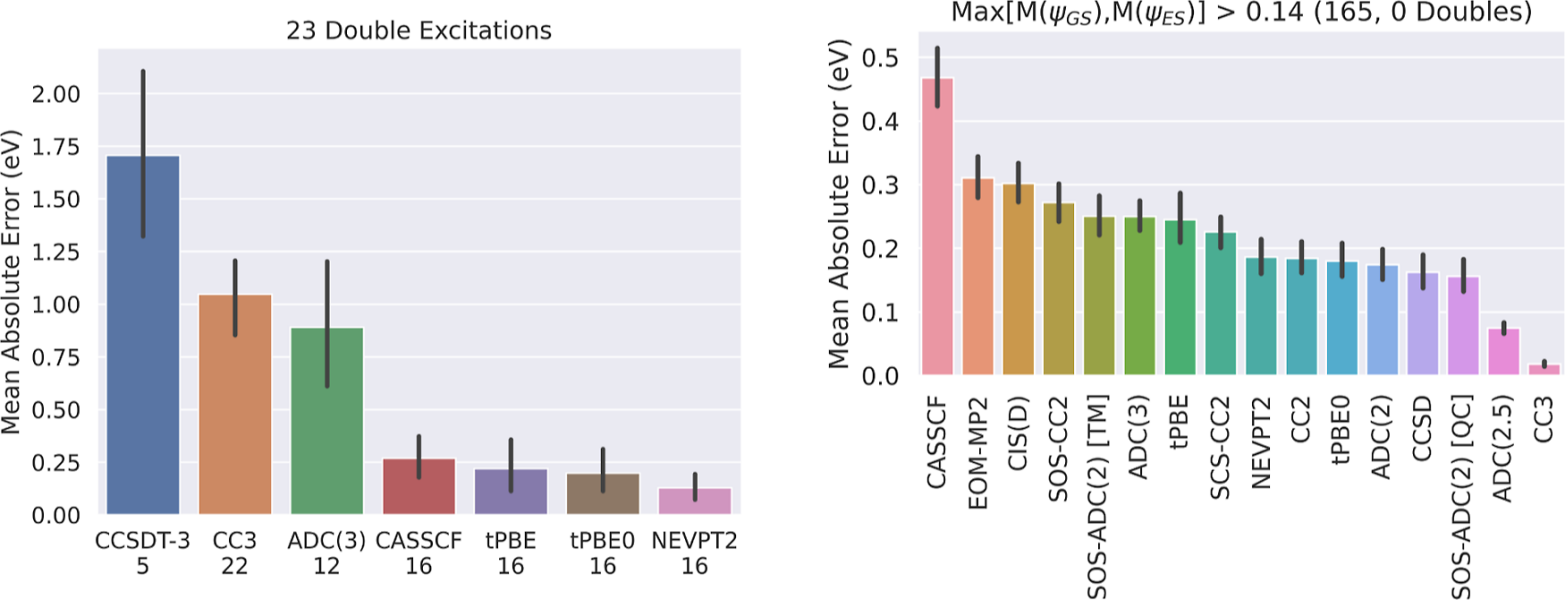
Left: Comparison of the mean absolute error of different methods on the entire subset of 23 double excitations in the QUESTDB data set. The amount of excitations available for each method (with SA-CASSCF, tPBE, tPBE0, and NEVPT2 included via a 1.1 eV SA-CASSCF error threshold) is marked under each bar. Right: Comparison of the mean absolute errors of various methods on the 165 Aug(12,12) excitations included by *T*_SA-CASSCF_ = 1.1 eV with high multireference character (Max[*M*(ψ_GS_),*M*(ψ_ES_)] > 0.14) and data available for every method shown, where *M* is the M diagnostic^40^ of the corresponding wave function. 95% confidence intervals are shown in black.

The left of Figure 5 shows the mean absolute error of different methods on excitations classified as double excitations for all methods with any calculated double excitations in the QUESTDB database; our automated approach was able to converge results within the 1.1 eV SA-CASSCF error threshold for 16/23 (70%) of the double excitations that have TBEs available, which is only slightly lower than the overall un-dropped-out fraction of 436/542 (80%) in the Aug(12,12) calculations. In keeping with the usual recommendation to use multireference methods for this class of excitation, we find that multireference methods are the only methods that perform consistently well on double excitations.

However, double excitations are not the only category of excitations, which are a challenge for single-reference approaches. To quantify the multireference character of single excitations, we have calculated the M diagnostic^40^ of the ground and excited state Aug(12,12) wave functions included by the 1.1 eV SA-CASSCF error threshold and use the maximum of these values, *M*_Max_ = Max[*M*(ψ_GS_),*M*(ψ_ES_)]. Doing so, we find that the lowest *M*_Max_ calculated for a double excitation is 0.14 (Supporting Information Table S12) and use this threshold as a classifier for identifying highly multireference single excitations; it happens to fall at slightly above the 50th percentile in the *M*_Max_ distribution (Supporting Information Figure S20). The right of Figure 5 shows a comparison of the mean absolute errors of SA-CASSCF, tPBE, tPBE0, and NEVPT2 to 12 single-reference methods on the 165 excitations with *M*_Max_ > 0.14 included by *T*_SA-CASSCF_ = 1.1 eV and data available for every method shown. Compared to Figure 5, we find that the performance of nearly all single-reference methods deteriorates significantly by about 0.05–0.07 eV when we consider only this high-*M*_Max_ subset; this brings the performance of CCSD into line with tPBE0.

In summary, we find tPBE0 and NEVPT2 to perform competitively on single excitations when compared to single-reference methods (Figures 4 and 5) and to be the only methods capable of reasonably describing double excitations (Figure 5). As such, we recommend tPBE0 and NEVPT2 as robust methods for calculating all classes of vertical excitations, although the active-space selection scheme may sometimes fail.

### 3.3 Performance by Excitation Type

**Figure 6:**
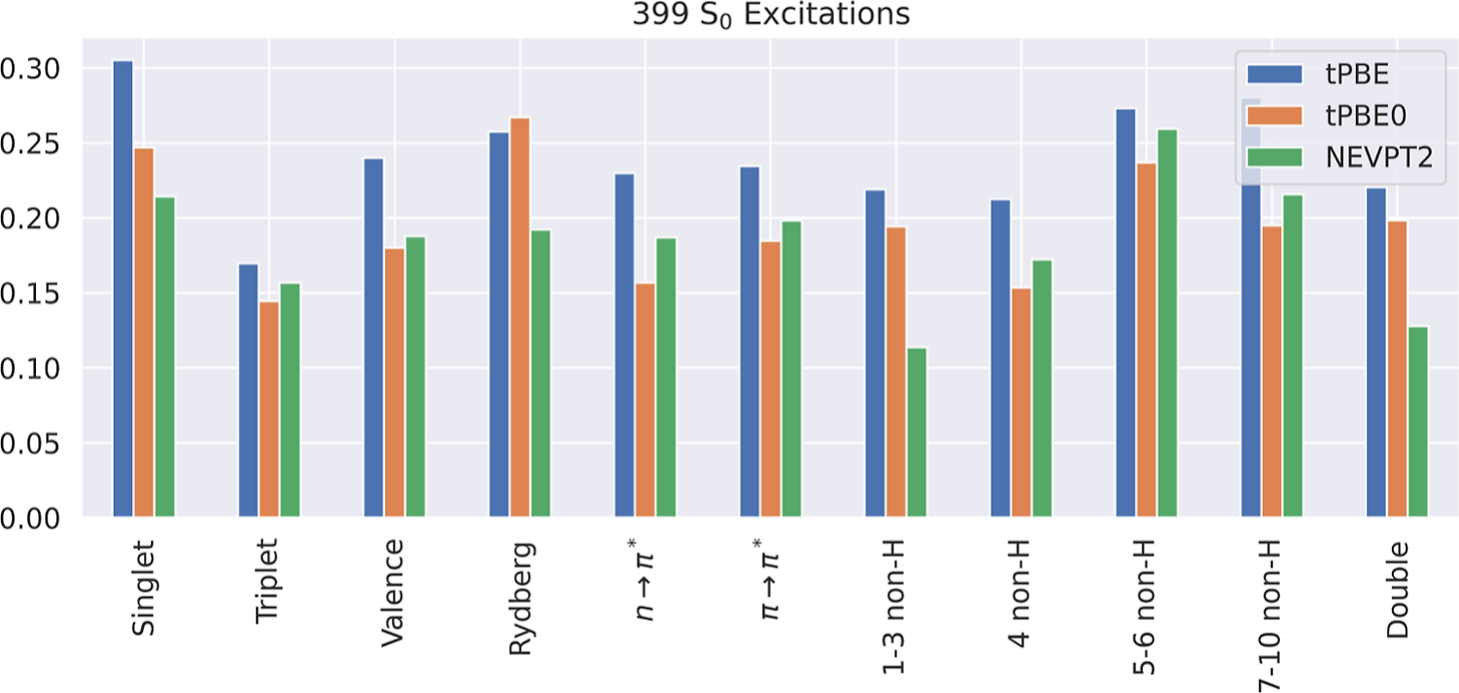
Mean absolute errors (in eV) of tPBE, tPBE0, and NEVPT2 Aug(12,12) calculations on various types of *S*_0_ excitations included by the threshold *T*_SA-CASSCF_ = 1.1 eV.

Figure 6 shows the errors classified by excitation type. In line with the Sarkar study,^84^ we find that NEVPT2 is more accurate for triplet excitations than singlet excitations, and tPBE and tPBE0 follow the same trend. The figure shows that, with the exception of Rydberg states, tPBE0 has better performance than tPBE for every excitation category, and therefore, we recommend the use of tPBE0 rather than tPBE for calculating excitation energies of valence excitations. We also recommend tPBE0 for calculating a spectrum containing both valence and Rydberg excitations since the performance of the two methods is very similar (on average) for Rydberg states.

### 3.4 Method Timing: tPBE0 Versus NEVPT2

**Figure 7:**
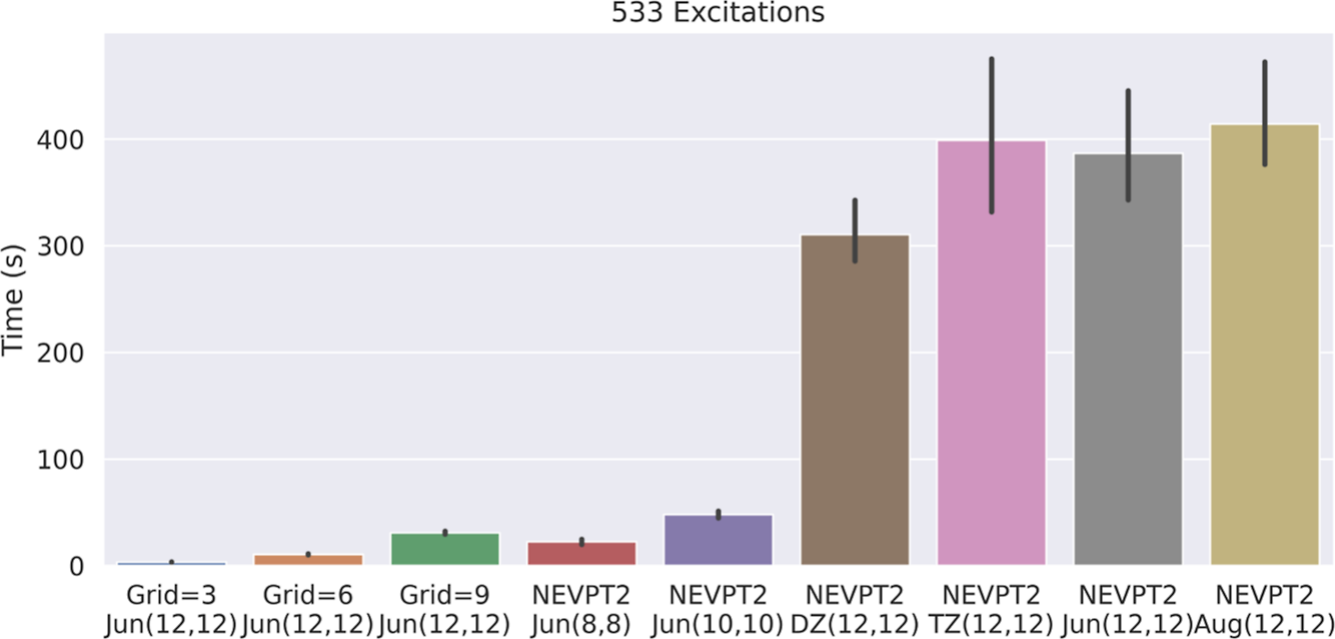
Comparison of the mean compute times for the post-SCF portion of tPBE calculations with various grid specifications and for the post-SCF portion of NEVPT2 calculations with various active spaces and basis set sizes on the set of 533 excitations that were converged with all active spaces and basis sets. The costs of the SA-CASSCF portions of the calculations were removed from these comparisons by caching the converged wave functions.

Figure 7 shows the average time consumed by the calculation of the NEVPT2 perturbative correction at different active space/basis set sizes and compares these timings to those for the calculation of the tPBE on-top energy by the methodology in Section 2. We find that at the normal grid size (grids_level = 3 in PySCF), tPBE is on average 114× less expensive than NEVPT2 for the large max(12,12) active spaces. This is because—as is well known—the cost of NEVPT2 scales very poorly with the size of the active space, while the cost of tPBE0 remains independent of that. Furthermore, the memory required for NEVPT2 also increases with active space size. It is around the max(12,12) active space size that the compute time for the perturbative correction begins to exceed the compute time of the underlying SA-CASSCF step, while the compute time of tPBE remains low.^86^ For smaller active spaces such as max(8,8), the cost of NEVPT2 is comparable to that of tPBE and tPBE0.

Keeping the cost down is important in many applications. Figure 7 shows that the dependence of MC-PDFT compute times on grid size is a significant consideration; we observe a roughly 10× increase in cost from grids_level = 3 (3.4 s) to grids_level = 9 (30.8 s). Our studies find that the standard grids_level = 3 in PySCF is sufficient for excitations such as those we have calculated because we only see a significant change between the maximum and default grid size for a single excitation (Supporting Information Figure S24). Therefore, we recommend standard grid sizes for most applications involving state-averaged MC-PDFT.

### 3.5 Method Timing: tPBE0 Versus CC2 and CCSD

**Figure 8:**
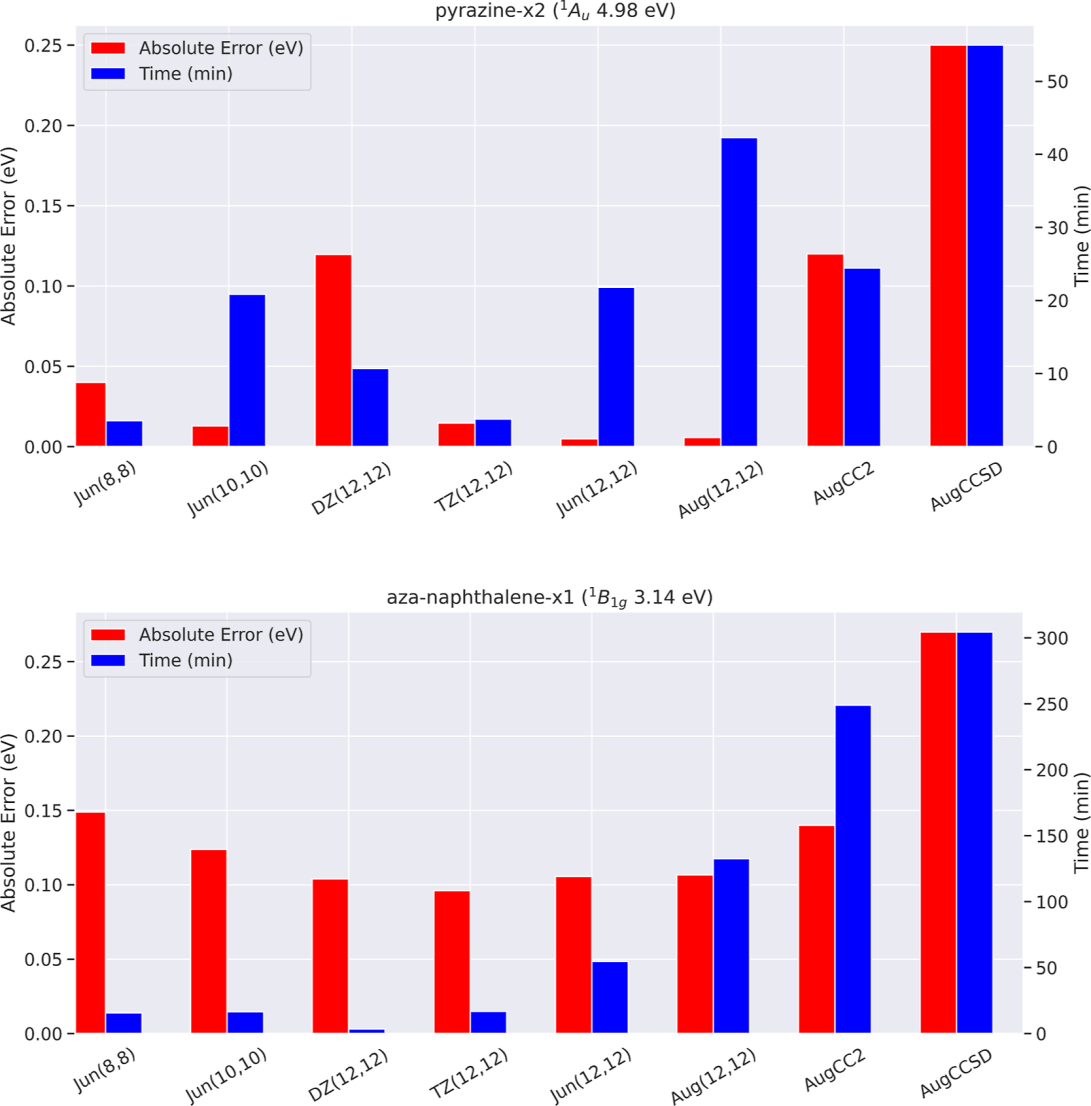
Comparison of timings and accuracy between tPBE0 at the six active space/basis set combinations explored in this work and CC2 and CCSD in the aug-cc-pVTZ basis. Timings for tPBE0 include the steps of RHF convergence, Boys orbital localization, active-space selection, CASSCF optimization, and computation of the tPBE0 nonclassical energy. Timings for CC2 and CCSD were computed in the aug-cc-pVTZ basis using their implementation in Psi4^87^ and were confirmed to reproduce the Jacquemin results.

In an effort to give greater context to the standing of tPBE0 as a method for calculating vertical excitations outside of cases where multireference methods are absolutely needed (such as double excitations), we compare the timings of complete CASSCF + tPBE0 calculations to those of CC2 and CCSD. Figure 8 shows the comparison of such timings for tPBE0 (including RHF convergence, Boys orbital localization, active-space selection, CASSCF optimization, and computation of the tPBE0 nonclassical energy) and CC2 and CCSD as computed in Psi4^87^ for two excitations in QUESTDB. All calculations were given the same amount of computational resources as outlined in Supporting Information Section S8.2. We have chosen to show timings and accuracies for both a “medium-sized” excitation (pyrazine-x2, with 368 aug-cc-pVTZ basis functions) and a “large-sized” excitation (aza-naphthalene-x1, with 552 aug-cc-pVTZ basis functions). Additionally, Figure 8 shows timings and accuracies for tPBE0 at all six of the active space and basis set combinations explored in this work.

Focusing first on the aug-cc-pVTZ calculations, one can see that tPBE0 takes a comparable amount of time compared to CC2 and CCSD, both for pyrazine and aza-naphthalene. However, in both of these cases, costs can be cut significantly, while maintaining accuracy by decreasing active space and basis set size. As demonstrated by Figure 8, through a judicious choice of active space and basis set, tPBE0 has the potential to be much less expensive than comparative single-reference approaches, while achieving similar accuracy or better. For aza-naphthalene-x1, tPBE0 is about 16× as fast as CC2 at Jun(8,8) and about 72× as fast at DZ(12,12). The speedup one can obtain tends to be greater when considering larger systems.

However, the idealized (albeit real) case shown for aza-naphthalene-x1 is far from general. First, one can only reduce basis set and active space size so far before one’s results become highly inaccurate with tPBE0, and the point at which this happens is highly excitation-dependent and somewhat dependent on the active-space selection scheme. Second, the timing behavior of CASSCF + tPBE0 is not always as well behaved: CASSCF optimization relies on a highly nonconvex and nonlinear optimization process, which may not conform to expected timing trends. An example of this can be seen in the pyrazine-x2 timings in Figure 8, where tPBE0@DZ(12,12) takes significantly more time than tPBE0@TZ(12,12). Further taking into account differences between implementations, we present Figure 8 only to give readers a rough sense of timings for tPBE0 with respect to comparably accurate single-reference methods on different system sizes.

Additionally, we attempted to compute timings for CC3 for these two excitations: the pyrazine-x2 result was computed in 1765 min (29 h) and aza-naphthalene-x1 was not able to finish within the 36 h time limit allowed by the resources available for these calculations. Finally, we note that CCSD also includes an iterative step, but a study of the convergence issues in CCSD is beyond our scope.

### 3.6 Optimizing the Mixing Parameter in Hybrid tPBE

A major motivation of this work was to generate data for benchmarking and improving MC-PDFT. As a first use of our data to optimize MC-PDFT functionals, we have investigated the optimal mixing parameter λ for hybrid tPBE (htPBE, for which the energy is given by λ*E*_SA-CASSCF_ + (1 – λ)*E*_tPBE_) over the Aug(12,12) database. We have chosen this set of excitations because it is likely to have the smallest amount of poor active spaces erroneously included by the *T*_SA-CASSCF_ = 1.1 eV error threshold. In other words, we expect this set of excitations to have the largest percentage of well-chosen active spaces.

**Figure 9:**
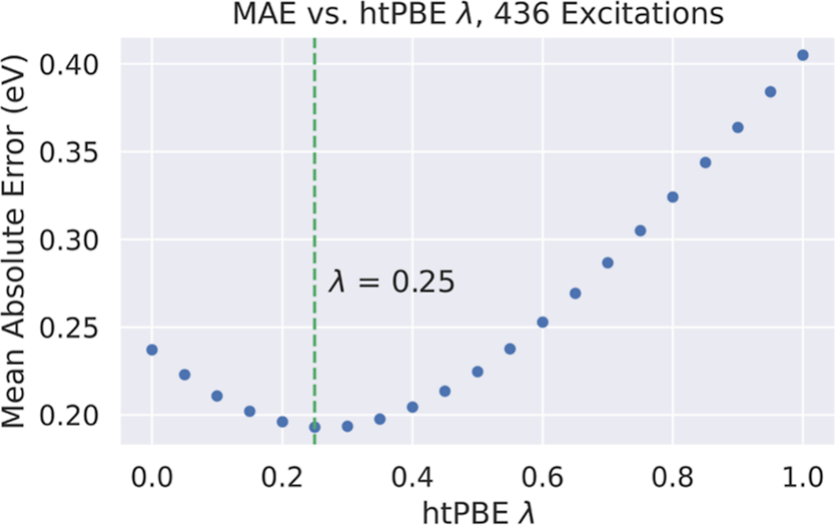
Mean absolute errors of different mixing parameters λ in energies computed by htPBE for the 436 Aug(12,12) excitations included with *T*_SA-CASSCF_ = 1.1 eV. The optimal value of λ = 0.25 (the same as in tPBE0) is marked with a dashed green line.

Figure 9 shows the optimization of λ on the Aug(12,12) set of included excitations. Delightfully, we find that λ = 0.25—the same parameter used in tPBE0—is optimal for this set of excitations, in agreement with the much smaller study previously conducted on the EE27 database.^37^ Therefore, we recommend using tPBE0 for excitation energies in the general case and especially for excitations similar to those in the QUESTDB data set. Optimizing the parameter over all active spaces and basis sets results in only a slightly shifted value of λ = 0.3, which appears to be offset mostly by the greater number of poor active spaces included in the Jun(8,8) excitation energies (Supporting Information Figure S22); using the more robust tPBE0 error threshold (discussed in the Supporting Information) removes this discrepancy (Supporting Information Figure S23). This suggests that a higher value of λ may be optimal for cases in which wave function error dominates.

## 4 Conclusions and Future Work

The work presented here is the largest application to date of automated multireference calculations on a broad range of molecules. The generation of 3237 multireference excitation energies has allowed us to gain insights into how to eliminate poorly chosen active spaces and has identified trends in the performance of MC-PDFT and NEVPT2. This work has been possible only through the careful work of Loos, Jacquemin, and co-workers in compiling the QUESTDB data set^20,62,64−66,88^ and the recent work of Sarkar et al.,^84^ which has enabled us to compare our automatically generated results to hand-selected active-space calculations.

We see this initial publication as laying the groundwork for several future applications related to MC-PDFT and high-throughput multireference calculations including

- Using the generated data to train and test novel functionals for MC-NCFT, representing a continuation of our initial work that used carbene singlet–triplet excitation energies to train machine-learned functionals.^36^
- Improving the active-space selection scheme. Our finding that error thresholds can be used to determine the fraction of poor wave functions in the calculated excitation energies can be used as a measure to benchmark the effectiveness of different active-space selection schemes.
- Determining if a selected active space is well chosen without reference to the underlying benchmark values. For specific active spaces and basis sets, there appears to be promise in looking at differences between different methods (Supporting Information Figure S13), but a method that is generalizable across active spaces and basis sets has yet to be found.

Additionally, we expect that the wave functions converged in this work will be of interest for the development of different post-CASSCF methods such as multireference adiabatic connection (AC)^89^ and algebraic diagrammatic construction (ADC).^90^ For this reason, we are making all 3237 converged wave functions freely available for public use. We hope that this data will be useful to the electronic structure community both for comparing to the results published here and for developing and testing their own methods.

In summary, we have carried out the largest benchmarking of SA-CASSCF and MC-PDFT to date. This was accomplished by means of an automatic active-space selection scheme and use of a SA-CASSCF error threshold to eliminate poor active-space choices. On a set of 373 aug-cc-pVTZ excitation energies, we find that tPBE0 and NEVPT2 perform with accuracy similar to CC2, while tPBE lags behind. However, the accuracy of NEVPT2 degrades with basis set size even as the quality of the underlying density and on-top pair density appear to remain the same. As expected, we find that tPBE0 is orders of magnitude less expensive than NEVPT2 for larger active spaces, and we recommend its use for the calculation of a broad range of excitation energies, including double excitations.

## Supporting Information Available

Analysis of N variation in APC-N, analysis of all 3237 excitation energies, tuning the error thresholds, reference-agnostic thresholds, analysis of Aug(12,12) data with *T_SA-CASSCF_* = 1.1 eV, comparison of Aug(12,12) excitations to other methods, alternative parameterizations of htPBE, and MC-PDFT grid size and timing. (PDF).
We have made available all the data necessary to reproduce and explore our results (orbitals, CI vectors, and metadata for all 3237 converged SA-CASSCF calculations) via Zenodo: https://doi.org/10.5281/zenodo.6644169
