# Supplementary material for: Large-Scale Benchmarking of Multireference Vertical-Excitation Calculations via Automated Active-Space Selection
Source: J Chem Theory Comput. 2022 Sep 16;18(10):6065–76. doi: 10.1021/acs.jctc.2c00630 (PMC9558375; doi:10.1021/acs.jctc.2c00630)
Supplement: Supplementary file 1 — ct2c00630_si_001.pdf [file ct2c00630_si_001.pdf]

Supporting Information

Large-Scale Benchmarking of Multireference Vertical-Excitation Calculations via Automated Active-Space Selection

Daniel S. King, Matthew R. Hermes, Donald G. Truhlar, Laura Gagliardi

September 15, 2022

Contents

1 Analysis of N Variation in APC-N **3**

1.1 Variation in Active Space Balance and SA-CASSCF Absolute Error 3

1.2 Variation of APC-N Entropies for Naphthalene 4

1.3 Definitions of Exchange and Fock Matrix Elements in APC 5

1.4 A Note on Entropy-Degenerate Orbital Ordering 6

2 Analysis of All 3237 Excitation Energies **7**

2.1 Percentage of Data Included by Different SA-CASSCF Error Thresholds 8

2.2 SA-CASSCF Summary Statistics 8

2.2.1 Without 1.1 eV SA-CASSCF Error Threshold ($T_{\text{SA-CASSCF}} = \infty$) 8

2.2.2 With 1.1 eV SA-CASSCF Error Threshold ($T_{\text{SA-CASSCF}} = 1.1$ eV) 9

2.2.3 Outlier Statistics Using $T_{\text{SA-CASSCF}} = 1.1$ eV 9

2.3 Effect of 1.1 eV SA-CASSCF Error Threshold on tPBE0 and NEVPT2 Errors 10

2.4 Distribution of Method Errors with $T_{\text{SA-CASSCF}} = 1.1$ eV 11

3 Tuning the Error Thresholds **12**

4 Reference-Agnostic Thresholds **18**

5 Analysis of Aug(12,12) Data With $T_{\text{SA-CASSCF}} = 1.1$ eV **20**

5.1 Data Included by System 21
5.2 Data Included by Method 22
5.3 Data Included by Type of Excitation 23
5.4 Summary Statistics of Included Excitations ($T_{SA-CASSCF}$ = 1.1 eV) 23
5.5 Summary Statistics of Excluded Excitations ($T_{SA-CASSCF}$ = 1.1 eV) 24
5.6 Performance on Doublet Excitations 24

6 Comparison of Aug(12,12) Excitations to Other Methods 24

6.1 Comparative Summary Statistics 25
6.2 Comparative Mean Absolute Error by Excitation Type 26
6.3 Comparison to Other Methods Using T_{NEVPT2} = 0.65 eV 27
6.4 Comparison to Other Methods Using T_{tPBE0} = 0.55 eV 28
6.5 Table of Double Excitations 29
6.6 Distribution of M Diagnostics 31
6.7 Comparison to Other Methods on Excitations with Low M Diagnostic 32

7 Alternative Parameterizations of htPBE 32

7.1 With 1.1 eV SA-CASSCF error threshold 33
7.2 With 0.55 eV tPBE0 Cutoff 33

8 MC-PDFT Grid Size and Timing 34

8.1 Maximum Deviations from Maximum Grid Fineness 34
8.2 Timing Methodology 34

1 Analysis of N Variation in APC-N

This section discusses the motivation behind the development of APC-N in which a dropout scheme is used on high-entropy virtual orbitals in calculating the orbital entropies (i.e. they are treated as singly occupied). This was done because highly correlated virtual orbitals tend to overcorrelate all the the doubly occupied orbitals, resulting in imbalanced active spaces. We find that much more balanced active spaces are selected at APC-2.

1.1 Variation in Active Space Balance and SA-CASSCF Absolute Error

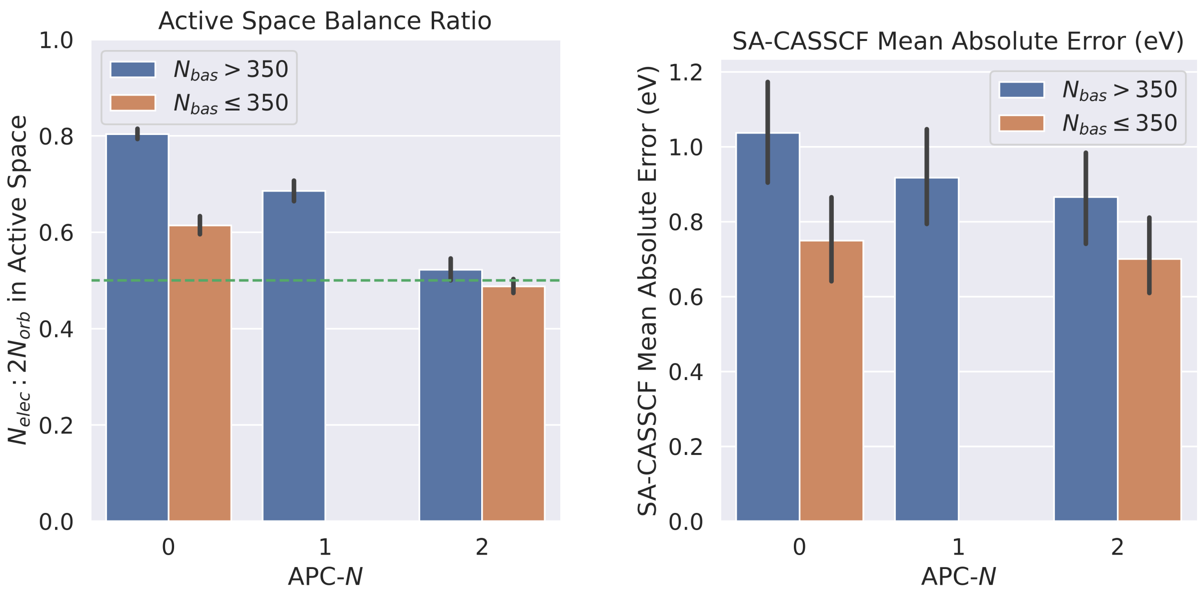

Figure 1: Variation in the active space balance and SA-CASSCF absolute error between APC-0, APC-1, and APC-2. Left: The ratio of the number of electrons in the active space N_{elec} to twice the number of orbitals in the active space, $2N_{orb}$. A balanced active space such as (10,10) or (12,12) will have a ratio of 0.5 while electron-heavy active spaces such as (18,10) will have a ratio greater than 0.5; this value is marked with a dashed green line. Right: The SA-CASSCF mean absolute error. Each category is split between a set of 167 excitations from larger molecules with greater than 350 aug-cc-pVTZ basis functions ($N_{bas} > 350$) and a set of 368 excitations from smaller molecules with less than 350 aug-cc-pVTZ basis functions ($N_{bas} \leq 350$). The effect of moving from APC-0 to APC-2 is seen much more strongly in larger systems than in smaller systems, although smaller systems do not appear to become imbalanced in the opposite direction as a result. Active spaces become more balanced and mean SA-CASSCF error decreases as APC-0 is changed to APC-2.

1.2 Variation of APC-N Entropies for Naphthalene

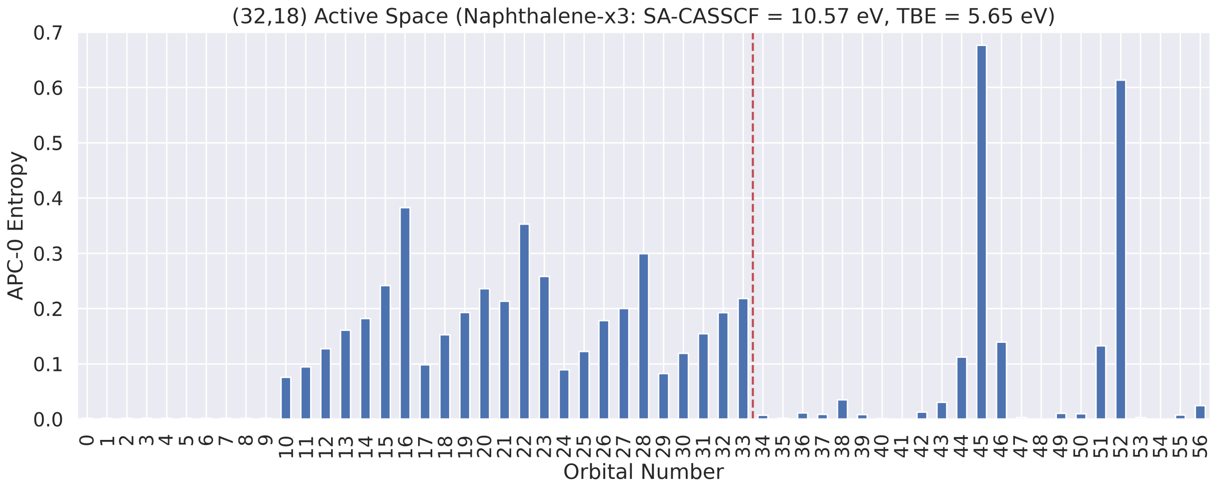

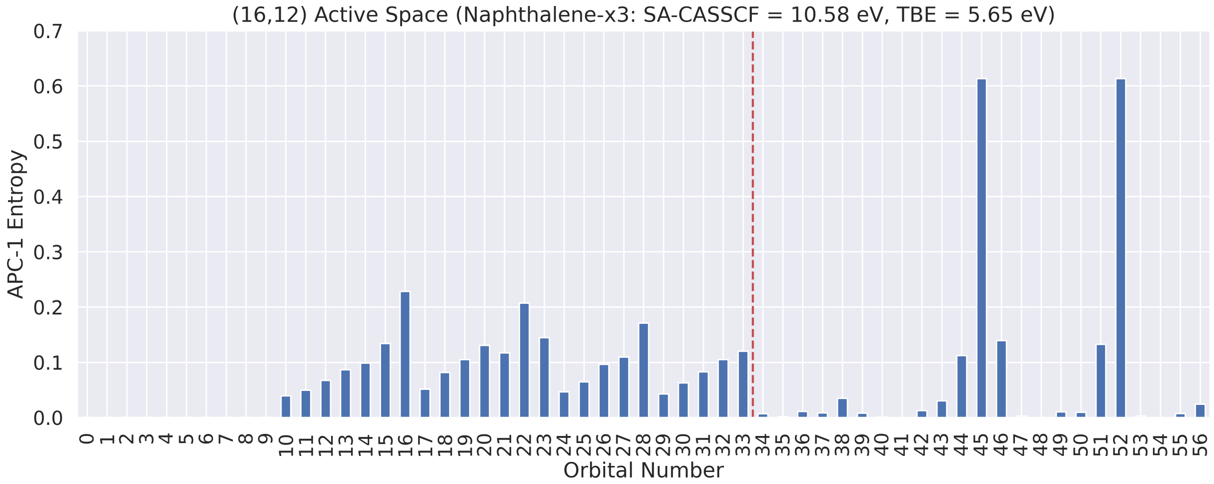

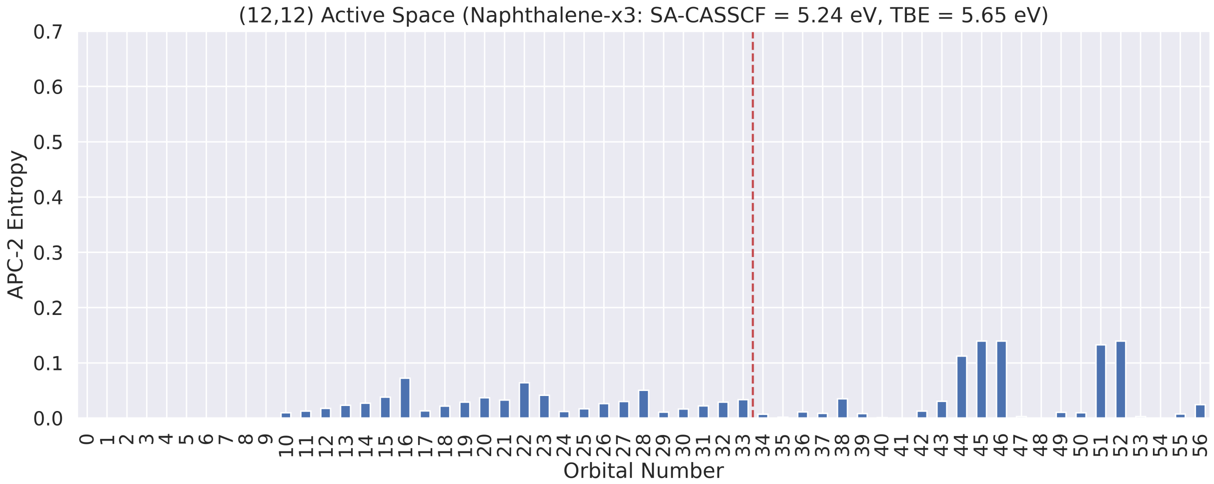

Figure 2: APC-N entropies for Boys-localized aug-cc-pVTZ naphthalene orbitals as N is varied from 0 to 2. The separating line between doubly occupied orbitals and virtual orbitals is drawn in red (naphthalene has 68 electrons, so orbitals from 0 to 33 are occupied, and orbital 33 is the HOMO). The overall magnitude of the doubly occupied entropies decreases as highly correlating orbitals are removed from the active space, and the entropy of the more weakly correlating virtual orbitals are put more in line with the entropy of the doubly occupied orbitals. Going from APC-0 to APC-2, the selected active space varies from (32,18) to (16,12) to (12,12), and the SA-CASSCF excitation energy naphthalene-x3 ($^1A_g \rightarrow {}^1B_{3u}$) is varied from 10.57 eV to 5.24 eV with respect to the theoretical best estimate (TBE) of 5.65 eV.

1.3 Definitions of Exchange and Fock Matrix Elements in APC

For a RHF wave function the Fock matrix (F) and exchange matrix (K) are given in the AO basis by

$$K_{uv} = \sum_{\lambda\sigma} \gamma_{\lambda\sigma} (u\lambda|v\sigma) \tag{1}$$

$$F_{uv} = H_{uv}^{core} + \sum_{\lambda\sigma} \gamma_{\lambda\sigma} (uv|\lambda\sigma) - 0.5 \sum_{\lambda\sigma} \gamma_{\lambda\sigma} (u\lambda|v\sigma) \tag{2}$$

where H_{uv}^{core} is a matrix element of the one-electron nuclear-electron attraction and the electronic kinetic energy operator, and γ_{ab} is the spin-summed density matrix in the AO basis. These matrices are then transformed to the candidate orbital basis via

$$K_{ij} = \sum_{uv} C_{ui} F_{uv} C_{vj} \tag{3}$$

$$F_{ij} = \sum_{uv} C_{ui} F_{uv} C_{vj} \tag{4}$$

where C_{ij} are the candidate MO coefficients. The relevant diagonal elements of these matrices F_{ii}, F_{aa}, and K_{aa} are those used in the APC algorithm for RHF.

In UHF and ROHF, the mean-field exchange felt by electrons differs by their spin, resulting in two spin-separated exchange matrices K^{α} and K^{β}:

$$K^{\alpha}_{uv} = \sum_{\lambda\sigma} \gamma^{\alpha}_{\lambda\sigma}(u\lambda|v\sigma) \tag{5}$$

$$K^{\beta}_{uv} = \sum_{\lambda\sigma} \gamma^{\beta}_{u\lambda\sigma}(u\lambda|v\sigma) \tag{6}$$

where $\gamma^{\alpha}_{\lambda\sigma}$ and $\gamma^{\beta}_{\lambda\sigma}$ are the spin-separated density matrices. In this case we simply sum the two exchange matrices together, which converges to the RHF limit when $K^{\alpha} = K^{\beta}$:

$$K = K^{\alpha} + K^{\beta} \tag{7}$$

There are also differences in the Fock matrix used in ROHF and UHF, as the exchange operator contributes to the Fock operator. For UHF, the spin-separated Fock matrices are given by

$$F^{\alpha}_{uv} = H^{core}_{uv} + \sum_{\lambda\sigma} \gamma_{\lambda\sigma}(uv|\lambda\sigma) - \sum_{\lambda\sigma} \gamma^{\alpha}_{\lambda\sigma}(u\lambda|v\sigma) \tag{8}$$

$$F^{\beta}_{uv} = H^{core}_{uv} + \sum_{\lambda\sigma} \gamma_{\lambda\sigma}(uv|\lambda\sigma) - \sum_{\lambda\sigma} \gamma^{\beta}_{\lambda\sigma}(u\lambda|v\sigma) \tag{9}$$

and for the APC scheme we simply average these together, which converges to the RHF limit:

$$F = \frac{1}{2}(F^{\alpha} + F^{\beta}) \tag{10}$$

In the ROHF case it is not as clear how to define the Fock matrix. Following PySCF defaults, we opt to use the Roothan effective Fock matrix for APC.[1–3]

1.4 A Note on Entropy-Degenerate Orbital Ordering

It is currently the case that the APC scheme can give slightly inconsistent active space selections as orbitals with identical entropies are dropped from the orbital array in a non-deterministic fashion. This entropy degeneracy can occur either because the orbitals are truly degenerate, having identical diagonal exchange and energy elements, or because the degeneracy has been imposed by the APC-N scheme by setting singly occupied and previously removed orbitals to the maximum value of the entropy array. While the former degeneracy is a somewhat inevitable consequence of the flexibility of APC scheme, the latter degeneracy can be removed by setting a standard entropy ordering of these orbitals. Currently the only well-defined behavior for these cases is that in the selection function any singly occupied orbitals are set to have a slightly larger

entropy (+0.01) than the remaining orbitals. However, as there could potentially be more than one singly occupied orbital, this can run into issues as well.

In future iterations of APC-N we will be attempting to define this behavior by setting the removed and singly occupied entropies in the following order. With the singly occupied orbitals ordered from lowest to highest in energy $\{o_1, o_2, ...\}$ and the removed orbitals ordered from first removed to last removed $\{r_1, r_2\}$, the entropies will be assigned with respect to the maximum element in the remaining orbitals S_{max} as

$$S(o_i) = S_{\mathrm{max}} + 2\delta - \eta(i-1) \tag{11}$$

$$S(r_i) = S_{\mathrm{max}} + \delta - \eta(i-1) \tag{12}$$

with $\eta/\delta << 1$ to affirm the ordering of singly occupied entropies over removed orbital entropies. Currently, all o_i and r_i have been set to S_{max}.

Note: During the writing of this section a small bug was found where the array of APC entropies was initialized as an empty array instead of an array of zeros. As such, r_i and o_i were sometimes set to $S > S_{\mathrm{max}}$ instead of S_{max} (which is actually more in line with future intended behavior). This error could have affected cases where one was deciding between throwing out a previously removed orbital or the maximum-entropy non-removed orbital. However, because all selected active spaces in APC-2 have at least 3 virtual orbitals, we do not believe this has impacted any conclusions.

2 Analysis of All 3237 Excitation Energies

In this section, we evaluate the summary statistics and error distributions of various methods with and without applying an SA-CASSCF error threshold. It is shown that applying $T_{\text{SA-CASSCF}} =$ 1.1 eV results in remarkably similar inlier and outlier distributions between active spaces and basis sets. Additionally we show that NEVPT2 and tPBE0 error are strongly correlated at high SA-CASSCF error, which provides further evidence that large errors are due to problems with the SA-CASSCF active space and not to the method of calculating the energy from it.

2.1 Percentage of Data Included by Different SA-CASSCF Error Thresholds

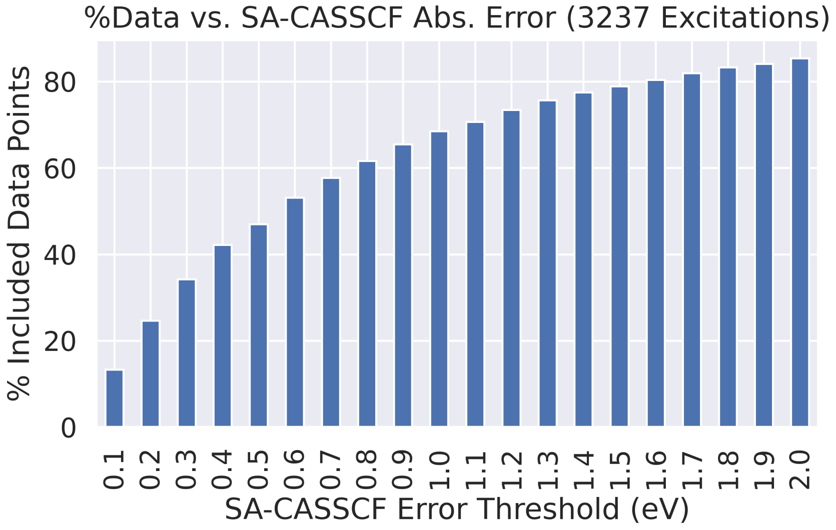

Figure 3: Percentage of included data of all 3237 excitation energies as the SA-CASSCF error threshold ($T_{\text{SA-CASSCF}}$) is increased.

2.2 SA-CASSCF Summary Statistics

2.2.1 Without 1.1 eV SA-CASSCF Error Threshold ($T_{\text{SA-CASSCF}} = \infty$)

Table 1: Summary statistics of SA-CASSCF for various active spaces and basis sets without an applied 1.1 eV SA-CASSCF error threshold ($T_{\text{SA-CASSCF}} = \infty$). The total error distributions are significantly different between active space and basis set sizes.

	Count	MSE	MAE	Med	RMSE	SDE	Max(+)	Max(-)	Over	Under
Aug(12,12)	540.0	0.45	0.75	0.46	1.22	1.13	5.55	-9.13	393	147
Jun(12,12)	541.0	0.57	0.79	0.44	1.31	1.18	5.34	-9.16	408	133
TZ(12,12)	541.0	1.02	1.16	0.65	1.83	1.52	8.85	-9.16	483	58
DZ(12,12)	541.0	1.22	1.35	0.75	2.10	1.71	9.63	-9.26	494	47
Jun(10,10)	537.0	0.70	0.96	0.48	1.59	1.43	9.92	-9.03	393	144
Jun(8,8)	537.0	0.96	1.21	0.55	1.99	1.75	10.50	-8.85	395	142

2.2.2 With 1.1 eV SA-CASSCF Error Threshold ($T_{\text{SA-CASSCF}}$ = 1.1 eV)

Table 2: Summary statistics of SA-CASSCF for various active spaces and basis sets on excitations included by $T_{\text{SA}-\text{CASSCF}}$ = 1.1 eV. The error distributions are remarkably similar between different active space and basis set sizes, with the only significant difference being that the smaller basis set sizes are shifted significantly more towards overestimation.

	Count	MSE	MAE	Med	RMSE	SDE	Max(+)	Max(-)	Over	Under
Aug(12,12)	436	0.18	0.40	0.34	0.50	0.47	1.10	-1.05	298	138
Jun(12,12)	428	0.22	0.38	0.31	0.48	0.43	1.08	-1.05	301	127
TZ(12,12)	350	0.35	0.40	0.33	0.50	0.36	1.08	-0.82	298	52
DZ(12,12)	327	0.37	0.42	0.33	0.51	0.35	1.10	-0.75	286	41
Jun(10,10)	383	0.14	0.35	0.28	0.45	0.43	1.08	-1.09	247	136
Jun(8,8)	363	0.16	0.37	0.29	0.47	0.45	1.09	-0.99	228	135

2.2.3 Outlier Statistics Using $T_{\text{SA-CASSCF}}$ = 1.1 eV

Table 3: Summary statistics of SA-CASSCF for various active spaces and basis sets on excitations excluded by $T_{\text{SA}-\text{CASSCF}}$ = 1.1 eV. The error distributions of the outliers are very similar between different active space and basis set sizes.

	Count	MSE	MAE	Med	RMSE	SDE	Max(+)	Max(-)	Over	Under
Aug(12,12)	104	1.59	2.21	1.70	2.58	2.03	5.55	-9.13	95	9
Jun(12,12)	113	1.86	2.37	1.90	2.70	1.97	5.34	-9.16	107	6
TZ(12,12)	191	2.25	2.55	1.84	3.01	2.00	8.85	-9.16	185	6
DZ(12,12)	214	2.51	2.78	2.09	3.28	2.10	9.63	-9.26	208	6
Jun(10,10)	154	2.07	2.47	1.90	2.88	2.01	9.92	-9.03	146	8
Jun(8,8)	174	2.62	2.96	2.47	3.43	2.22	10.50	-8.85	167	7

2.3 Effect of 1.1 eV SA-CASSCF Error Threshold on tPBE0 and NEVPT2 Errors

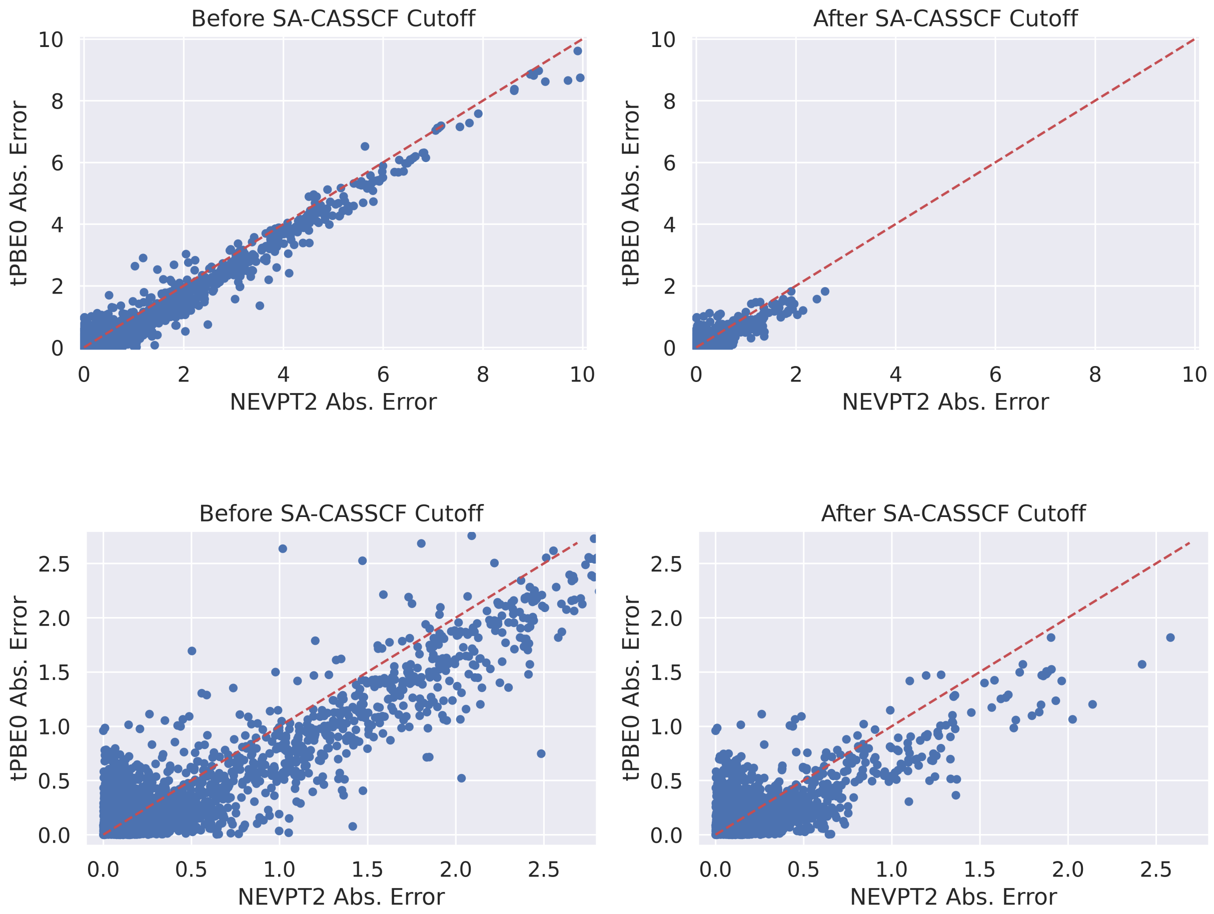

Figure 4: Top: Comparison of the tPBE0 and NEVPT2 errors of all 3237 converged excitations across active space and basis set variations before and after applying the SA-CASSCF error threshold. Bottom: The same as the top but with the ranges of the axes decreased to better fit the data remaining after applying the SA-CASSCF error threshold. Applying the SA-CASSCF error threshold decreases the maximum tPBE0 absolute error from 9.61 to 1.82 eV and the maximum NEVPT2 error from 9.95 to 2.58 eV. Surprisingly, tPBE0 is consistently about 0.23 eV better than NEVPT2 on outlier active spaces, even as errors approach 6-9 eV. While tPBE0 and NEVPT2 have similar errors on data included by the SA-CASSCF error threshold (0.20 eV vs. 0.22 eV), their errors diverge significantly on data not included in the SA-CASSCF error threshold (1.75 vs. 1.98 eV).

2.4 Distribution of Method Errors with $T_{\text{SA-CASSCF}} = 1.1$ eV

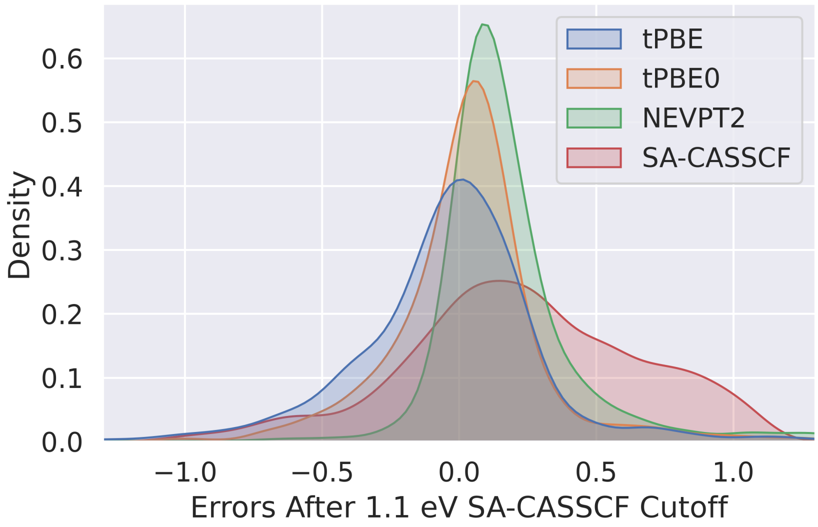

Figure 5: Kernel density estimations of SA-CASSCF, tPBE, tPBE0, and NEVPT2 errors on all 2287 included excitations after applying the $T_{\text{SA-CASSCF}} = 1.1$ eV. Trends can be seen such as the routine overestimation of NEVPT2 and the significant improvement of tPBE0 over tPBE via cancellation of error with SA-CASSCF.

3 Tuning the Error Thresholds

The main text shows how the use of a 1.1 eV threshold for the SA-CASSCF error results in intuitive trends and reasonable errors for tPBE0 and NEVPT2. The discussion here provides justification for this particular value of the SA-CASSCF threshold by showing how this value is optimal for reproducing the curated multireference results of Sarkar et al.,[4] in addition to suggesting alternative thresholds for tPBE0 and NEVPT2.

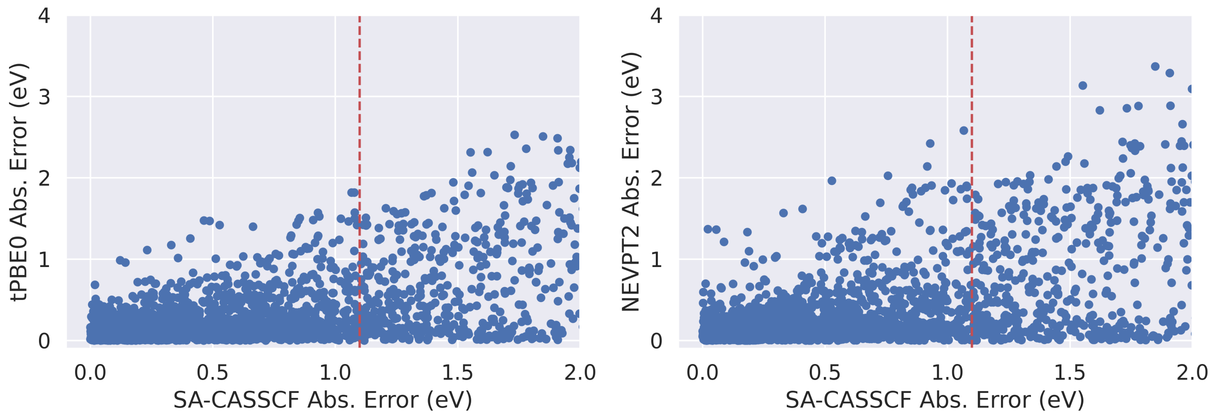

Figure 6: Scatter plots of tPBE0 and NEVPT2 errors on excitations with SA-CASSCF error less than 2 eV; a dashed red line demarcating $T_{\mathrm{SA-CASSCF}} = 1.1$ eV is shown in each plot.

To begin, we note that from a simple visual analysis of the data there is no clear choice for selecting $T_{\mathrm{SA-CASSCF}}$. Figure 7 shows how the SA-CASSCF absolute error is a more-or-less continuous measure of active space quality, with the average tPBE0 and NEVPT2 error continually decreasing as $T_{\mathrm{SA-CASSCF}}$ is decreased. For example, applying $T_{\mathrm{SA-CASSCF}} = 0.1$ eV results in tPBE0 and NEVPT2 mean absolute errors of 0.11 and 0.13 eV, respectively (albeit on only 13% of the converged data, SI Figure 3). Thus, it is clear that some trade-off must be made between including too little (and unrealistically good) active spaces and including too many (and unrealistically bad) active spaces. In other words, the included calculations should represent a "realistic" active space quality one might converge by carefully choosing the active space by hand.

Recently, Sarkar et al. have submitted CASPT2 and NEVPT2 results in the aug-cc-pVTZ basis for a subset of 35 small and medium closed-shell organic molecules in the QUESTDB database.[4] Because these active spaces were selected by hand, these results give us a good sense of what should count as a "reasonable" wave function result in non-automated practical applications. Sarkar et al. report summary statistics for a set of 265 aug-cc-pVTZ excitations,[4] of which we were able to calculate 255. This difference comes from 3 factors: adiabatic excitations, excitations included in the Sarkar dataset but not in the supporting information of Véril et al.,[5] and unconverged SA-CASSCF calculations.

Adiabatic Excitations. There are 5 adiabatic excitations reported by Sarkar et al. which we have not reproduced:

- $^1A''$ state in cyanoacetylene (3.54 eV)
- $^1\Sigma_u^-$ state in cyanogen (5.05 eV)
- $^1A''$ state in diazomethane (0.71 eV)
- $^1A''$ state in ketene (1.00 eV)
- $^1A''$ state in nitrosomethane (1.67 eV)

Missing Excitations in Véril et al. There are 3 excitations in Sarkar et al. that are absent in the excel sheet available in the supporting information of Véril et al. which was used in this work.[5]

- $^1A''$ state in imidazole (6.71 eV)
- $^1A'$ state in imidazole (6.86 eV)
- $^1A'$ state in imidazole (7.00 eV)

Unconverged Excitations. There are 2 excitations in this set that did not converge (most likely due to a bad active space choice):

- $^1B_1$ state in pyridazine (3.83 eV)
- $^1A_2$ state in pyridazine (4.37 eV)

Additionally there appears to be inconsistent rounding (e.g., 5.70 vs. 5.71) on many excitations between Véril et al.[5] and Sarkar et al..[4] Despite this, the effects of the missing excitations as well as this rounding error are very small and we are comfortable comparing to the statistics given by Sarkar et al. using our dataset of 255 excitations (about 50 of which are excluded by applied error thresholds).

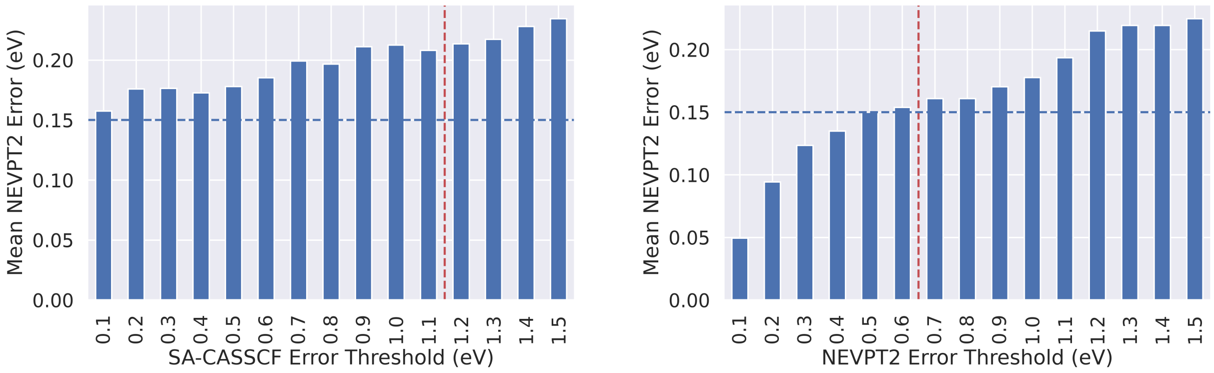

Figure 7: Mean absolute NEVPT2 errors of excitations included by varying SA-CASSCF and NEVPT2 error thresholds on our subset of 255 excitations also calculated by Sarkar et al.[4] The Sarkar MAE for NEVPT2 of 0.15 eV is dotted in blue, and $T_{\text{SA-CASSCF}} = 1.1$ eV and $T_{\text{NEVPT2}} = 0.65$ eV are dotted in red.

Figure 7 shows the mean absolute error for NEVPT2 we obtain on our automated reproduction of the Sarkar dataset when applying increasingly tight SA-CASSCF and NEVPT2 error thresholds. A SA-CASSCF error threshold as low as $T_{\mathrm{SA-CASSCF}}$ = 0.08 eV is required to achieve the 0.15 eV MAE for NEVPT2 reported by Sarkar et al.[4] In contrast, thresholding on NEVPT2 achieves a NEVPT2 MAE of 0.15 eV with a cutoff of only T_{NEVPT2} = 0.61 eV, which is consistent with the maximum NEVPT2 error observed in the Sarkar study of 0.65 eV.[4]

Table 4: Summary statistics of SA-CASSCF, tPBE, tPBE0, and (strongly contracted) NEVPT2 on our 210 excitations included by T_{NEVPT2} = 0.65 eV on our Sarkar dataset of excitaitons, compared to the summary statistics of 265 excitations for SC-NEVPT2 published by Sarkar et al.[4]

	Count	MSE	MAE	RMSE	SDE	Max(+)	Max(-)
SA-CASSCF	210.0	0.22	0.52	0.66	0.62	2.01	-1.05
Sarkar SA-CASSCF	265.0	0.12	0.47	0.59	0.56	2.14	-1.18
NEVPT2	210.0	0.14	0.16	0.20	0.15	0.63	-0.33
Sarkar NEVPT2	265.0	0.13	0.15	0.19	0.13	0.65	-0.38
tPBE	210.0	-0.09	0.21	0.30	0.28	0.41	-1.56
tPBE0	210.0	-0.02	0.16	0.22	0.22	0.54	-1.11

Table 5: Mean absolute errors of SA-CASSCF, tPBE, tPBE0, and (strongly contracted) NEVPT2 by excitation type on our 210 excitations included by T_{NEVPT2} = 0.65 eV on the Sarkar dataset of excitaitons, compared to the summary statistics by excitation type of 265 excitations for SC-NEVPT2 published by Sarkar et al.

	SA-CASSCF	Sarkar SA-CASSCF	NEVPT2	Sarkar NEVPT2	tPBE	tPBE0
Singlet	0.61	0.55	0.18	0.16	0.27	0.20
Triplet	0.41	0.34	0.14	0.13	0.15	0.11
Valence	0.52	0.44	0.16	0.15	0.21	0.13
Rydberg	0.52	0.53	0.16	0.15	0.22	0.25
$n \to \pi^*$	0.68	0.44	0.12	0.12	0.21	0.10
$\pi \to \pi^*$	0.41	0.44	0.18	0.17	0.22	0.15
Double	0.15	0.16	0.07	0.04	0.20	0.16

Indeed, applying a NEVPT2 cutoff to our data equal to the maximum error observed in the Sarkar study, T_{NEVPT2} = 0.65 eV, reproduces the summary statistics of Sarkar et al. remarkably well (Tables 4 and 5). Thus, these results show that multireference results curated by hand can be reproduced by the APC scheme with use of a proper error threshold.

Inspired by the results above, we chose to take the maximum-Sarkar-error threshold of T_{NEVPT2} = 0.65 eV as a benchmark in order to tune error thresholds for CASSCF and tPBE0. A standard way to optimize threshold classifiers is by analysis of the receiver operating characteristic (ROC) curve, which plots the percentage of true positives identified (in this case, poor active spaces

identified as such) against the percentage of false positives identified (in this case, good active spaces identified as poor) as the threshold is increased:[6]

$$\mathrm{TP}(T) = \frac{|\{\Psi : (\Delta\Delta E_T > T) \wedge (\Delta\Delta E_{\mathrm{NEVPT2}} > 0.65)\}|}{|\{\Psi : \Delta\Delta E_{\mathrm{NEVPT2}} > 0.65\}|} \tag{13}$$

$$\mathrm{FP}(T) = \frac{|\{\Psi : (\Delta\Delta E_T > T) \wedge (\Delta\Delta E_{\mathrm{NEVPT2}} < 0.65)\}|}{|\{\Psi : \Delta\Delta E_{\mathrm{NEVPT2}} < 0.65\}|} \tag{14}$$

where T is some error threshold classifier, $\Delta\Delta E_T$ is the error type being thresholded, and $\Delta\Delta E_{\mathrm{NEVPT2}}$ is the NEVPT2 error. In words, $\mathrm{TP}(T)$ is equal to the number of calculations for which both $\Delta\Delta E_T > T$ and $\Delta E_{\mathrm{NEVPT2}} > 0.65$ divided by the total number of calculations for which $E_{\mathrm{NEVPT2}} > 0.65$, and $\mathrm{FP}(T)$ is equal to the number of calculations for which $\Delta\Delta E_T > T$ but $\Delta E_{\mathrm{NEVPT2}} < 0.65$ divided by the total number of calculations for which $\Delta E_{\mathrm{NEVPT2}} < 0.65$.

Thus, each point in ROC space ($\mathrm{FP}(T)$ vs. $\mathrm{TP}(T)$) represents the performance characteristics for a single choice of T. ROC analysis is particularly attractive for this problem because it is insensitive to changes in the class distribution (in this case, poor and reasonable active spaces)[6] which will vary significantly between different active spaces and basis sets.

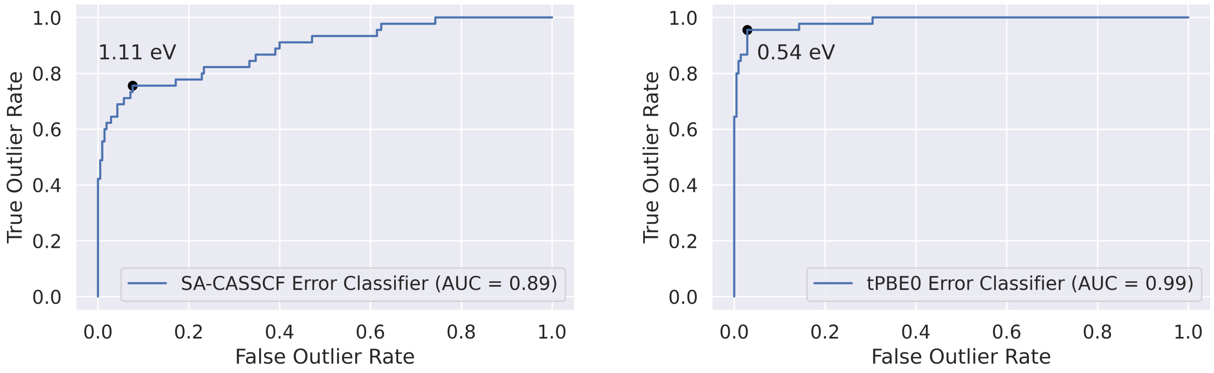

Figure 8: Receiver operating characteristic curves of SA-CASSCF and tPBE0 error threshold classifiers in identifying calculations in our 255-excitation Sarkar dataset with NEVPT2 error less than 0.65 eV. The optimal value maximizing the Youden's J statistic[7] is found to be 1.11 eV for SA-CASSCF and 0.54 eV for tPBE0.

Figure 8 shows the ROC curves for the SA-CASSCF error and tPBE0 error classifiers, along with the reported area under the ROC curve (AUC-ROC) which represents the quality of the classifier in discriminating between classes. A perfect classifier will include all true positives before including any false positives and thus have an AUC-ROC of 1, while a random classifier will include true positives and false positives at equal rate and have an AUC-ROC of 0.5. The optimal thresholds for a classifier can be found by choosing the point closest to (0,1) by optimizing the Youden's J statistic.[7] We find that the optimal value for SA-CASSCF is 1.11 eV and for tPBE0 is 0.54 eV; we round these to the nearest 0.05 eV to avoid overfitting.

Table 6: Summary statistics of SA-CASSCF, tPBE, tPBE0, and (strongly contracted) NEVPT2 on our 205 excitations included by $T_{\text{SA-CASSCF}}$ = 1.1 eV on the Sarkar dataset of excitations, compared to the summary statistics of 265 excitations for SC-NEVPT2 published by Sarkar et al.[4]

	Count	MSE	MAE	RMSE	SDE	Max(+)	Max(-)
SA-CASSCF	205.0	0.14	0.45	0.54	0.53	1.09	-1.05
Sarkar SA-CASSCF	265.0	0.12	0.47	0.59	0.56	2.14	-1.18
NEVPT2	205.0	0.19	0.21	0.33	0.28	1.66	-0.69
Sarkar NEVPT2	265.0	0.13	0.15	0.19	0.13	0.65	-0.38
tPBE	205.0	-0.03	0.24	0.36	0.36	1.64	-1.56
tPBE0	205.0	0.02	0.19	0.30	0.30	1.40	-1.11

Table 7: Summary statistics of SA-CASSCF, tPBE, tPBE0, and (strongly contracted) NEVPT2 on our 208 excitations included by T_{tPBE0} = 0.55 eV on the Sarkar dataset of excitaitons, compared to the summary statistics of 265 excitations for SC-NEVPT2 published by Sarkar et al.[4]

	Count	MSE	MAE	RMSE	SDE	Max(+)	Max(-)
SA-CASSCF	208.0	0.24	0.52	0.66	0.62	2.01	-1.05
Sarkar SA-CASSCF	265.0	0.12	0.47	0.59	0.56	2.14	-1.18
NEVPT2	208.0	0.15	0.17	0.24	0.18	1.10	-0.69
Sarkar NEVPT2	265.0	0.13	0.15	0.19	0.13	0.65	-0.38
tPBE	208.0	-0.07	0.20	0.27	0.26	0.53	-0.96
tPBE0	208.0	0.01	0.15	0.19	0.19	0.54	-0.54

Tables 6 and 7 show the performances of the optimized CASSCF and tPBE0 error thresholds in reproducing the Sarkar summary statistics. As one can see, the tPBE0 threshold does a much better job of reproducing the Sarkar results in accordance with its better AUC score (0.99 vs. 0.87). Given these results, we emphasize that the SA-CASSCF error threshold of $T_{\text{SA-CASSCF}}$ = 1.1 eV is by no means perfect at eliminating poor active spaces; this is the reason for the slight inhomogeneities between active spaces and basis sets (especially going to Jun(8,8)) observed in Manuscript Figure 2: as the active space and basis set sizes are decreased the proportion of poor active spaces increases in turn and thus more poor active spaces are included in the data by $T_{\text{SA-CASSCF}}$ = 1.1 eV. However, given our analysis we believe this choice of threshold to be optimal, and differences in summary statistics largely come from only a small amount of outlier active spaces.

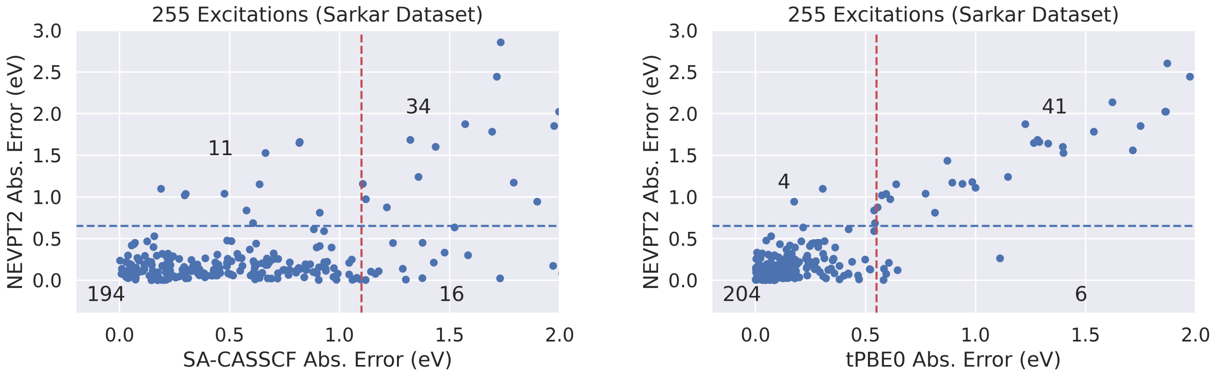

Figure 9: Scatter plot confusion matrices showing the performance of the ROC-optimized $T_{\text{SA}-\text{CASSCF}} = 0.65$ (left) and ROC-optimized $T_{\text{tPBE0}} = 0.55$ eV (right) in predicting a NEVPT2 error less than 0.65 eV. The ROC-optimized thresholds are shown in red and the 0.65 eV NEVPT2 threshold is shown in blue.

Figure 9 shows the plotted confusion matrices of the optimized SA-CASSCF and tPBE0 error classifiers in predicting NEVPT2 errors less than 0.65 eV. As one can see, both classifiers are fairly good at eliminating poor NEVPT2 calculations (89% accuracy for $T_{\text{SA-CASSCF}} = 1.1$ eV and 96% accuracy for $T_{\text{tPBE0}} = 0.55$ eV), but with tPBE0 having significantly better performance. It is the fault of only the 11/205 calculations included by the 1.1 eV SA-CASSCF error threshold with high NEVPT2 error that causes the SC-NEVPT2 statistics to deviate significantly from those of Sarkar et al. (Table 6).

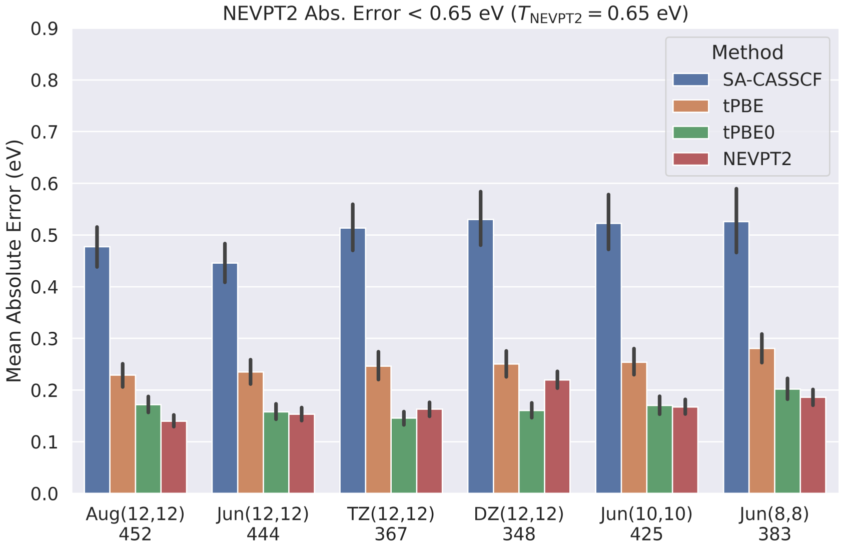

Figure 10: Mean absolute errors of SA-CASSCF, tPBE, tPBE0, and NEVPT2 calculations for various active spaces and basis sets on excitations included by $T_{\text{NEVPT2}} = 0.65$ eV. Using this threshold, tPBE0 achieves an absolute error of 0.17 ± 0.019 eV. A similar amount of calculations are included at each active space and basis set compared to $T_{\text{SA-CASSCF}} = 1.1$ eV.

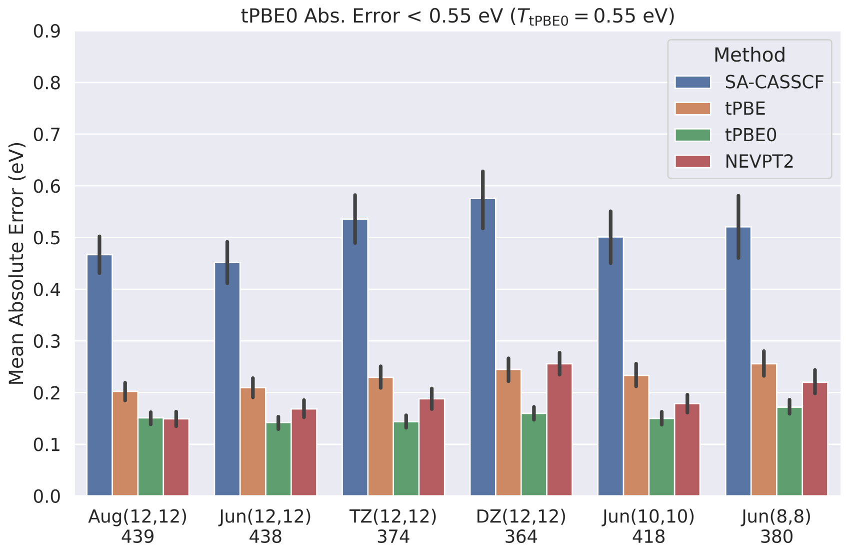

Figure 11: Mean absolute errors of SA-CASSCF, tPBE, tPBE0, and NEVPT2 calculations for various active spaces and basis sets on excitations included by $T_{\text{tPBE0}} = 0.55$ eV. Using this threshold, tPBE0 achieves an absolute error of 0.15 ± 0.011 eV. A similar amount of calculations are included at each active space and basis set compared to $T_{\text{SA-CASSCF}} = 1.1$ eV.

Figures 10 and 11 show the performances of SA-CASSCF, tPBE, tPBE0, and NEVPT2 using the reference cutoff of $T_{\text{NEVPT2}} = 0.65$ eV and the optimized tPBE0 cutoff of $T_{\text{tPBE0}} = 0.55$ eV, respectively. Along with Figure 2 in the main text, these plots show that all 3 cutoffs are generalizable between different active spaces and basis sets. However, the performance of tPBE0 is dependent on the threshold used, achieving an error of 0.17 ± 0.019 eV with $T_{\text{NEVPT2}} = 0.65$ and an error of 0.15 ± 0.011 eV with $T_{\text{tPBE0}} = 0.55$ eV, in contrast to the error of 0.20 ± 0.02 eV with $T_{\text{SA-CASSCF}} = 1.1$ eV reported in the main text.

We believe that the 0.15 and 0.17 eV error numbers are likely closer to what one would achieve carefully selecting active spaces by hand as we believe the tPBE0 and NEVPT2 thresholds to be more reliable classifiers for determining poor active spaces. This greater reliability is also reflected in the more consistent behavior with λ in tuning htPBE (SI section 7). Despite this, we have decided to exclusively use the SA-CASSCF error threshold in the main text as it unbiased towards any particular post-CASSCF method. However, if one wishes to use this data for other purposes we suggest using the tPBE0 error threshold as it appears to have better performance, and tPBE0 error is consistent across different basis set sizes (as long as the wave functions remain well-described) unlike NEVPT2.

4 Reference-Agnostic Thresholds

Despite the success of using thresholds with respect to benchmark values to eliminate poor active spaces, developing "reference-agnostic" thresholds – ones that can be applied without a reference in hand – is obviously of great interest. In this section, we present our attempts

at developing thresholds on several reference-agnostic criteria such as $|\Delta E_{\mathrm{tPBE0}} - \Delta E_{\mathrm{SA-CASSCF}}|$, $|\Delta E_{\mathrm{NEVPT2}} - \Delta E_{\mathrm{SA-CASSCF}}|$, and $|\Delta E_{\mathrm{NEVPT2}} - \Delta E_{\mathrm{tPBE0}}|$.

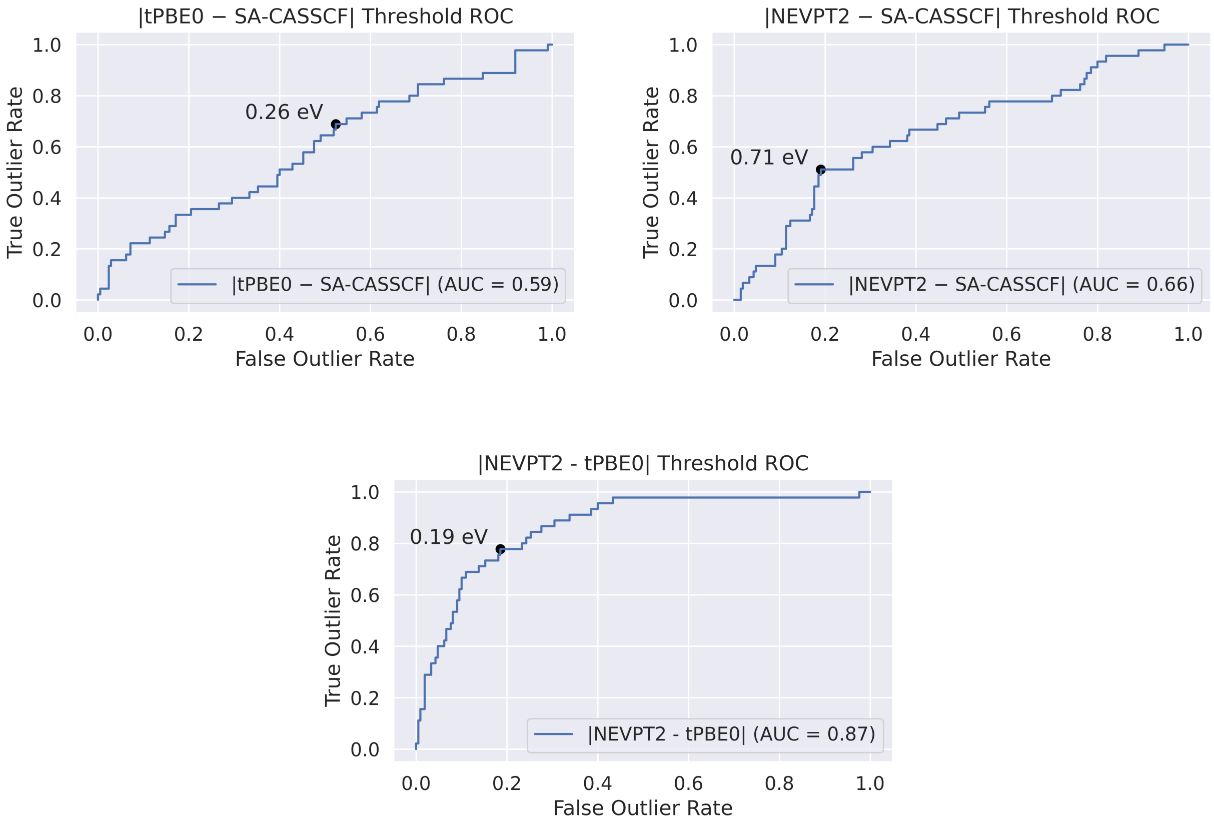

Figure 12: ROC analyses for various different threshold classifiers used to predict $T_{\mathrm{NEVPT2}} = 0.65$ eV, including the absolute difference between tPBE0 and SA-CASSCF, the absolute difference between NEVPT2 and SA-CASSCF, the absolute difference between NEVPT2 and tPBE0, and the tPBE0 absolute error vs. the theoretical best estimate.

Figure 12 shows the ROC curves for these different classifiers. Of these three options we find that $|\Delta E_{\mathrm{NEVPT2}} - \Delta E_{\mathrm{tPBE0}}|$ serves as the best classifier with an AUC of 0.87 and optimized threshold of 0.19 eV, which is comparable to the SA-CASSCF error AUC of 0.89.

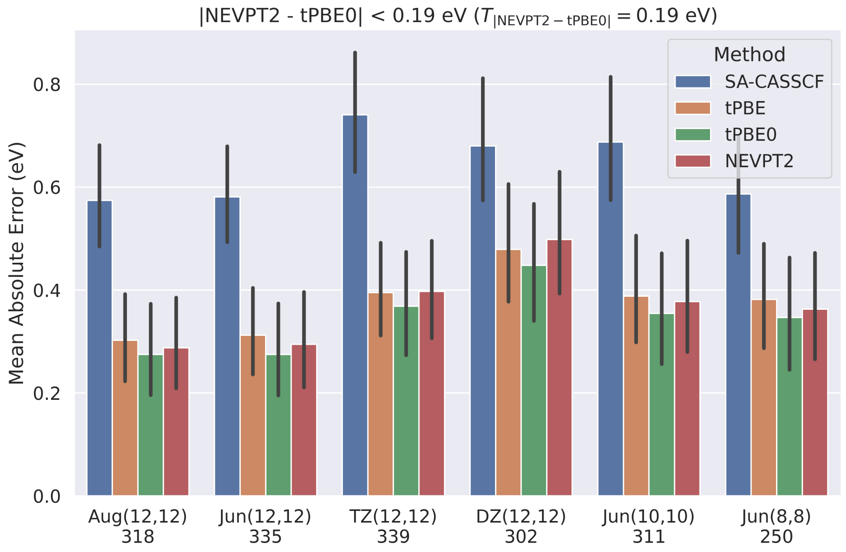

Figure 13: Mean absolute errors of SA-CASSCF, tPBE, tPBE0, and NEVPT2 calculations for various active spaces and basis sets on excitations included by $T_{|\mathrm{tPBE0-NEVPT2}|} = 0.19$ eV.

However, Figure 13 shows how $T_{|\mathrm{tPBE0-NEVPT2}|} = 0.19$ eV does not appear to be generalizable across different active spaces and basis sets, perhaps being due to to the inconsistent performance of NEVPT2 as discussed in the main text.

As such, we are currently without a totally satisfactory reference-agnostic classifier, and this may be a future direction for research. Nevertheless, the main text suggests that comparing SA-CASSCF to one's best available estimate and using the 1.1 eV threshold that works reasonably well when the best estimate is accurate may serve as a useful working criterion for a good active space.

5 Analysis of Aug(12,12) Data With $T_{\text{SA-CASSCF}} = 1.1$ eV

In this section, we discuss the data availability and summary statistics of our Aug(12,12) results for SA-CASSCF, tPBE, tPBE0, and NEVPT2 on the entire QUESTDB set of 542 excitations on excitations included by $T_{\text{SA-CASSCF}} = 1.1$ eV.

5.1 Data Included by System

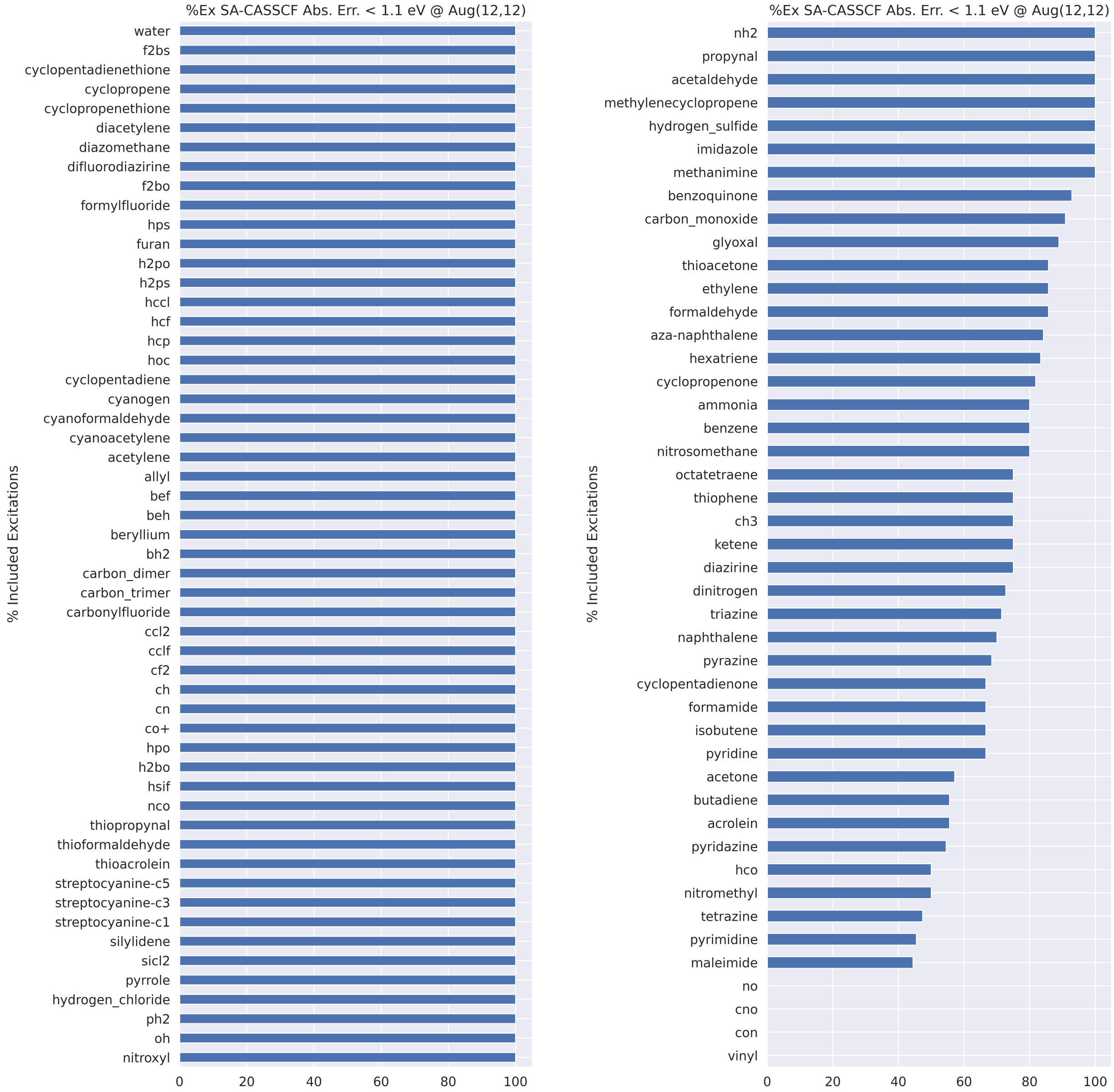

Figure 14: Percent of SA-CASSCF excitations calculated at the Aug(12,12) level satisfying the 1.1 eV SA-CASSCF error threshold. All but four of the 99 systems (vinyl, CON, CNO, and NO) have at least one excitation included in the threshold; we suspect that for these systems we may have obtained an incorrect geometry from the SI of Véril et al.[5] due to unit conversion. Overall 65/99 systems have all excitations included in the threshold with an average inclusion of 86% of excitations per system. This number differs only slightly from the total number of included excitations (436/542 = 80.4%) due to the fact that larger more difficult systems are weighted more heavily in terms of their total number of excitations.

5.2 Data Included by Method

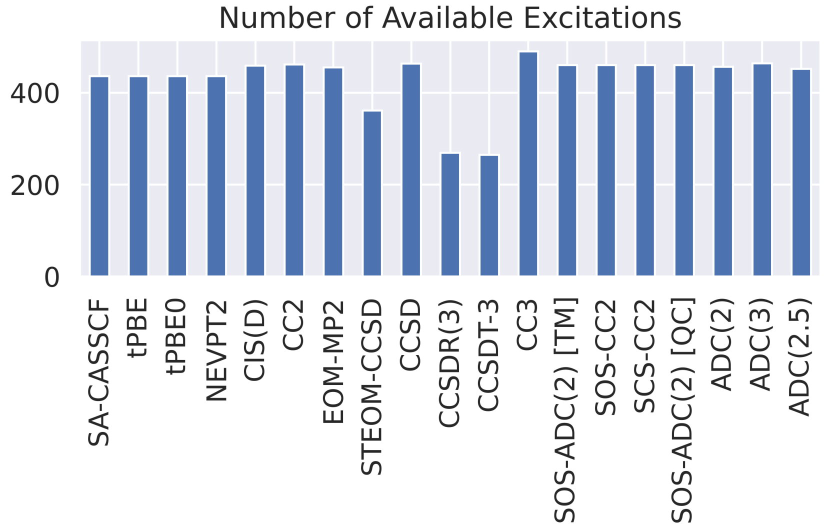

Figure 15: Total number of excitations available for QUESTDB by method, with results from other methods taken from the tabulated results in the SI of the QUESTDB publication.[5] Although the percentage of calculations excluded by $T_{\text{SA-CASSCF}}$ = 1.1 eV is significant, (1 - 436/542 = 19.6%), a comparable number of excitations are available for SA-CASSCF, tPBE, tPBE0, and NEVPT2 due to their computation on on doublet and double excitations (79 included by $T_{\text{SA-CASSCF}}$ = 1.1 eV) for which values for most of the other tabulated methods are not generally available (although results are available on other methods for about half of the unsafe excitations). To obtain the consistent set of 373 excitations analyzed in the main manuscript, STEOM-CCSD, CCSDR(3), and CCSDT-3 were excluded from analysis.

5.3 Data Included by Type of Excitation

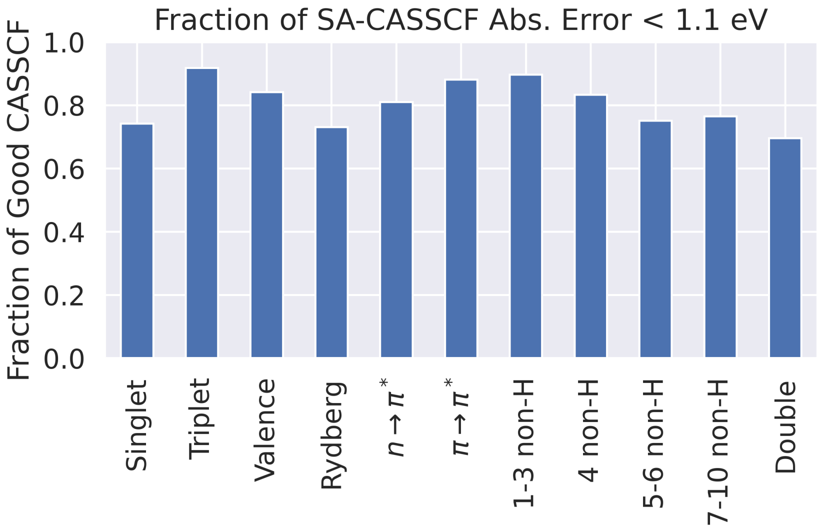

Figure 16: Fractions of excitations included by $T_{\text{SA-CASSCF}}$ = 1.1 eV by excitation type. There is no noticeable drop in inclusion for any specific type of excitation.

5.4 Summary Statistics of Included Excitations ($T_{\text{SA−CASSCF}} = 1.1$ eV)

Table 8: Summary statistics of SA-CASSCF, tPBE, tPBE0, and NEVPT2 on the 436 Aug(12,12) excitations included by $T_{\text{SA−CASSCF}} = 1.1$ eV.

	MSE	MAE	Med	RMSE	SDE	Max(+)	Max(-)	Over	Under
SA-CASSCF	0.18	0.40	0.34	0.50	0.47	1.10	-1.05	298	138
tPBE	-0.08	0.24	0.16	0.35	0.34	1.64	-1.64	174	262
tPBE0	-0.02	0.19	0.13	0.29	0.29	1.40	-1.11	221	215
NEVPT2	0.15	0.18	0.11	0.30	0.27	1.84	-0.69	361	75

5.5 Summary Statistics of Excluded Excitations ($T_{\text{SA-CASSCF}} = 1.1$ eV)

Table 9: Summary statistics of SA-CASSCF, tPBE, tPBE0, and NEVPT2 on the 436 Aug(12,12) excitations excluded by $T_{\text{SA-CASSCF}} = 1.1$ eV. Large SA-CASSCF overestimations (95) are vastly more common than large SA-CASSCF underestimations (9).

	MSE	MAE	Med	RMSE	SDE	Max(+)	Max(-)	Over	Under
SA-CASSCF	1.59	2.21	1.70	2.58	2.03	5.55	-9.13	95	9
tPBE	0.60	1.54	1.08	2.20	2.12	6.12	-8.76	61	43
tPBE0	0.85	1.54	1.14	2.22	2.06	5.98	-8.86	84	20
NEVPT2	1.10	1.72	1.22	2.41	2.16	6.48	-8.95	81	23

5.6 Performance on Doublet Excitations

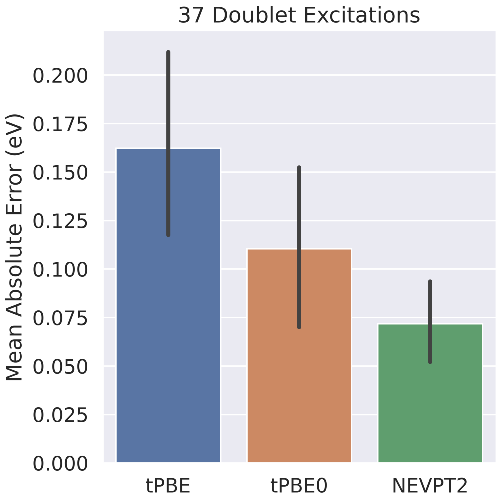

Figure 17: Comparison of tPBE, tPBE0, and NEVPT2 on the 37 doublet excitations included at Aug(12,12) using $T_{\text{SA-CASSCF}} = 1.1$ eV.

6 Comparison of Aug(12,12) Excitations to Other Methods

In this section, we discuss the comparison of our Aug(12,12) results to the aug-cc-pVTZ results reported for other methods in the QUESTDB database. We find that tPBE0 and NEVPT2 have comparable performance to CC2 with mean absolute errors of about 0.18 eV. Additionally we report comparisons using different error thresholds ($T_{\text{tPBE0}} = 0.55$ eV and $T_{\text{NEVPT2}} = 0.65$ eV), which complement the results shown for $T_{\text{SA-CASSCF}} = 1.1$ eV in the main text.

Additionally, we provide a complete list of the data available for double excitations in the QUESTDB database complemented by our results for SA-CASSCF, tPBE, tPBE0, and NEVPT2, as well as calculated M diagnostics for our calculations. Finally, we provide an analysis of the distribution of

M diagnostics within the Aug(12,12) excitations and provide a comparison to other methods on excitations with lower M diagnostic which complements the comparsion on excitations with high M diagnostics in the manuscript.

6.1 Comparative Summary Statistics

Table 10: Comparison of summary statistics on the 373 Aug(12,12) excitations included by $T_{\mathrm{SA-CASSCF}} = 1.1$ eV and with data available for all other methods shown.

	MSE	MAE	Med	RMSE	SDE	Max(+)	Max(-)	Over	Under
SA-CASSCF	0.18	0.42	0.37	0.52	0.48	1.10	-1.05	252	121
tPBE	-0.08	0.24	0.16	0.35	0.34	1.64	-1.64	161	212
tPBE0	-0.02	0.19	0.13	0.28	0.28	1.40	-1.11	198	175
NEVPT2	0.15	0.18	0.11	0.29	0.25	1.66	-0.69	314	59
CIS(D)	0.17	0.23	0.20	0.30	0.25	1.46	-0.64	307	58
CC2	0.06	0.15	0.11	0.21	0.20	0.90	-0.71	231	129
EOM-MP2	0.19	0.23	0.19	0.30	0.23	1.26	-0.38	280	85
CCSD	0.09	0.12	0.08	0.18	0.15	1.13	-0.25	278	86
CC3	0.00	0.02	0.01	0.03	0.03	0.19	-0.09	134	132
SOS-ADC(2) [TM]	0.19	0.21	0.17	0.27	0.19	1.40	-0.29	323	45
SOS-CC2	0.21	0.23	0.19	0.28	0.18	1.38	-0.24	342	29
SCS-CC2	0.16	0.19	0.17	0.24	0.17	1.22	-0.92	340	29
SOS-ADC(2) [QC]	0.03	0.13	0.09	0.19	0.19	1.18	-0.46	210	152
ADC(2)	0.02	0.15	0.11	0.21	0.21	0.97	-0.76	210	151
ADC(3)	-0.14	0.22	0.21	0.26	0.21	0.49	-1.01	75	297
ADC(2.5)	-0.06	0.08	0.07	0.10	0.08	0.19	-0.34	63	305

6.2 Comparative Mean Absolute Error by Excitation Type

Table 11: Comparison of the mean absolute error for different excitation types in the 373 Aug(12,12) excitations included by $T_{\mathrm{SA-CASSCF}}$ = 1.1 eV and with data available for all other methods shown.

	SA-CASSCF	tPBE	tPBE0	NEVPT2	CIS(D)	CC2	EOM-MP2	CCSD
Singlet	0.50	0.30	0.24	0.20	0.23	0.15	0.27	0.16
Triplet	0.34	0.17	0.14	0.15	0.23	0.16	0.19	0.08
Valence	0.41	0.23	0.17	0.18	0.25	0.15	0.25	0.13
Rydberg	0.47	0.26	0.27	0.18	0.18	0.18	0.16	0.07
$n \rightarrow \pi^*$	0.51	0.21	0.14	0.15	0.19	0.08	0.28	0.17
$\pi \rightarrow \pi^*$	0.37	0.23	0.18	0.19	0.29	0.20	0.24	0.12
1-3 non-H	0.36	0.21	0.19	0.11	0.21	0.19	0.13	0.07
4 non-H	0.33	0.19	0.13	0.13	0.22	0.18	0.15	0.12
5-6 non-H	0.50	0.28	0.24	0.25	0.23	0.12	0.29	0.13
7-10 non-H	0.55	0.28	0.20	0.22	0.30	0.12	0.46	0.23

	CC3	SOS-ADC(2) [TM]	SOS-CC2	SCS-CC2	SOS-ADC(2) [QC]
Singlet	0.02	0.22	0.24	0.19	0.16
Triplet	0.01	0.20	0.22	0.19	0.11
Valence	0.02	0.22	0.26	0.21	0.13
Rydberg	0.02	0.19	0.15	0.14	0.15
$n \rightarrow \pi^*$	0.01	0.27	0.33	0.23	0.12
$\pi \rightarrow \pi^*$	0.02	0.20	0.22	0.21	0.13
1-3 non-H	0.02	0.17	0.19	0.18	0.14
4 non-H	0.02	0.20	0.27	0.23	0.12
5-6 non-H	0.01	0.22	0.21	0.16	0.12
7-10 non-H	0.02	0.30	0.32	0.23	0.16

	ADC(2)	ADC(3)	ADC(2.5)
Singlet	0.15	0.20	0.08
Triplet	0.15	0.23	0.08
Valence	0.14	0.23	0.08
Rydberg	0.20	0.17	0.09
$n \rightarrow \pi^*$	0.09	0.15	0.07
$\pi \rightarrow \pi^*$	0.17	0.28	0.08
1-3 non-H	0.19	0.24	0.10
4 non-H	0.16	0.22	0.07
5-6 non-H	0.12	0.20	0.07
7-10 non-H	0.13	0.20	0.07

6.3 Comparison to Other Methods Using $T_{\mathrm{NEVPT2}} = 0.65$ eV

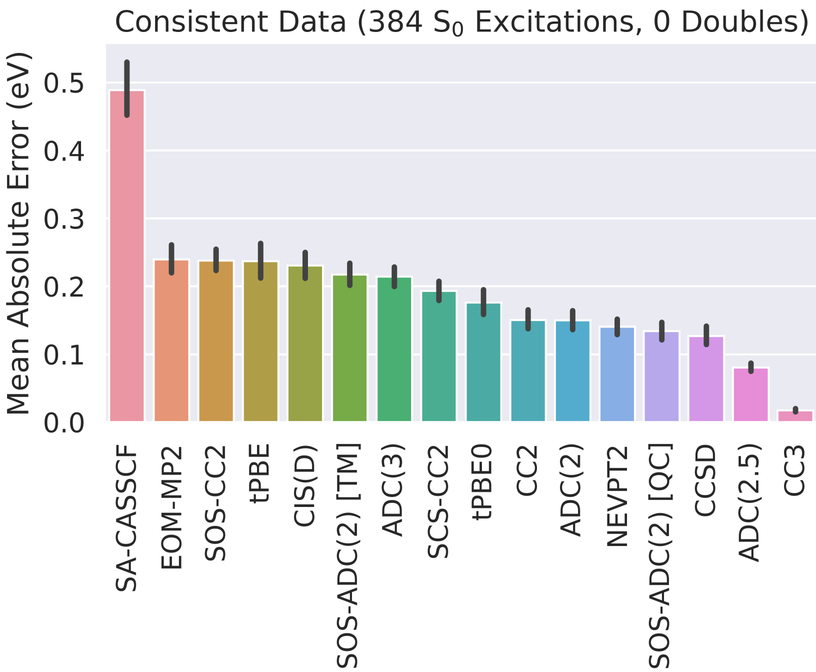

Figure 18: MAE comparison of the 384 Aug(12,12) excitations included by $T_{\mathrm{NEVPT2}} = 0.65$ eV with data available for all other methods shown. 95% confidence intervals are shown in black. A similar number of excitations are included compared to $T_{\mathrm{SA-CASSCF}} = 1.1$ eV (373).

6.4 Comparison to Other Methods Using $T_{\mathrm{tPBE0}} = 0.55$ eV

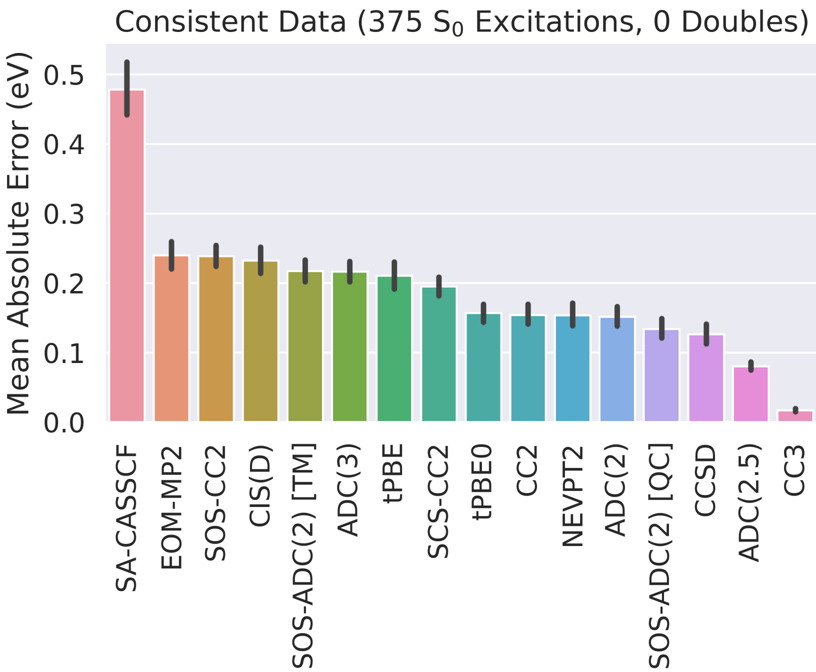

Figure 19: MAE comparison of the 375 Aug(12,12) excitations included by $T_{\mathrm{tPBE0}} = 0.55$ eV with data available for all other methods shown. 95% confidence intervals are shown in black. A similar number of excitations are included compared to $T_{\mathrm{SA-CASSCF}} = 1.1$ eV (373).

6.5 Table of Double Excitations

Table 12: Table of calculated double excitations, with SA-CASSCF, tPBE0, and NEVPT2 calculated at Aug(12,12) and excluded if the SA-CASSCF absolute error exceeded 1.1 eV. All available data from other methods is taken from the supporting information of QUESTDB.[5] The available data for each method is shown at the bottom of the table, along with the method used to calculate the TBE. The excitations are sorted by decreasing percentage of single excitations in CC3 (% T_1). Additionally we provide calculated M diagnostics for the ground and excited states of these double excitations;[8] M_{Max} gives the maximum of these values.

Name	SA-CASSCF	tPBE	tPBE0	NEVPT2	CCSDT-3	ADC(3)	CC3	TBE
acrolein-x5	-	-	-	-	-	-	8.08	7.87
cyclopentadienethione-x5	5.47	5.22	5.28	5.45	-	4.19	5.89	5.43
cyclopentadienone-x4	5.98	5.85	5.88	6.03	-	4.59	7.10	6.00
beryllium-x1	6.77	6.48	6.55	6.77	-	-	7.17	7.15
ethylene-x4	-	-	-	-	-	-	13.42	12.92
pyrazine-x12	-	-	-	-	-	-	9.17	8.04
cyclopentadienone-x9	5.12	4.95	4.99	5.01	-	4.30	6.05	4.91
tetrazine-x18	-	-	-	-	-	6.34	7.35	5.51
formaldehyde-x8	9.93	9.38	9.52	10.07	-	-	11.20	10.35
cyclopentadienethione-x9	3.64	3.20	3.31	3.16	-	2.35	4.39	3.13
cyclopentadienone-x3	5.74	4.95	5.14	5.04	-	4.37	6.12	5.02
nitrosomethane-x2	-	-	-	-	6.02	3.00	5.76	4.76
cyclopentadienethione-x3	3.27	3.21	3.23	3.22	-	2.34	4.40	3.16
carbon_trimer-x2	5.66	5.95	5.88	5.96	-	-	7.24	5.91
carbon_trimer-x1	5.18	5.42	5.36	5.34	-	-	6.68	5.22
tetrazine-x3	-	-	-	-	6.77	4.54	6.21	4.61
tetrazine-x8	-	-	-	-	-	6.54	7.62	6.15
glyoxal-x3	5.43	5.26	5.30	5.56	7.26	5.26	6.76	5.61
nitroxyl-x2	4.74	4.07	4.24	4.38	5.51	2.55	5.26	4.33
benzoquinone-x3	4.65	4.45	4.50	4.78	6.85	-	6.02	4.57
carbon_dimer-x1	2.55	2.06	2.18	2.30	-	-	3.05	2.09
carbon_dimer-x2	2.50	2.14	2.23	2.39	-	-	3.26	2.42
benzene-x7	10.12	10.55	10.44	10.96	-	-	-	10.55
MAE	0.27	0.22	0.20	0.13	1.71	0.89	1.05	-
MSE	0.06	-0.17	-0.11	0.04	1.71	-0.69	1.05	-
Count	16.00	16.00	16.00	16.00	5.00	12.00	22.00	23.00

name	TBE	TBE Method	T1	M(ψ_{GS})	M(ψ_{ES})	M_{Max}
acrolein-x5	7.87	exFCI/6-31+G(d) + [CC3/AVTZ - CC3/6-31+G(d)]	75.0	-	-	-
cyclopentadienethione-x5	5.43	NEVPT2/AVTZ	51.7	0.14	0.78	0.78
cyclopentadienone-x4	6.00	NEVPT2/AVTZ	49.9	0.13	0.65	0.65
beryllium-x1	7.15	exFCI/AVTZ	32.0	0.12	0.86	0.86
ethylene-x4	12.92	exFCI/AVTZ	20	-	-	-
pyrazine-x12	8.04	NEVPT2/AVTZ	12	-	-	-
cyclopentadienone-x9	4.91	NEVPT2/AVTZ	10	0.13	0.16	0.16
tetrazine-x18	5.51	NEVPT2/AVTZ	5.7	-	-	-
formaldehyde-x8	10.35	exFCI/AVTZ	5	0.07	0.38	0.38
cyclopentadienethione-x9	3.13	NEVPT2/AVTZ	4.2	0.15	0.16	0.16
cyclopentadienone-x3	5.02	NEVPT2/AVTZ	3.1	0.13	0.17	0.17
nitrosomethane-x2	4.76	exFCI/AVTZ	2.5	-	-	-
cyclopentadienethione-x3	3.16	NEVPT2/AVTZ	1.1	0.15	0.14	0.15
carbon_trimer-x2	5.91	exFCI/AVTZ	1	0.11	0.82	0.82
carbon_trimer-x1	5.22	exFCI/AVTZ	1	0.11	0.73	0.73
tetrazine-x3	4.61	NEVPT2/AVTZ	0.7	-	-	-
tetrazine-x8	6.15	NEVPT2/AVTZ	0.7	-	-	-
glyoxal-x3	5.61	exFCI/AVDZ + [CCSDT/AVTZ + - CCSDT/AVDZ]	0.5	0.10	0.52	0.52
nitroxyl-x2	4.33	exFCI/AVTZ	0.3	0.09	0.14	0.14
benzoquinone-x3	4.57	NEVPT2/AVTZ	0.04	0.14	0.96	0.96
carbon_dimer-x1	2.09	exFCI/AVTZ	0	0.39	0.16	0.39
carbon_dimer-x2	2.42	exFCI/AVTZ	0	0.38	0.27	0.38
benzene-x7	10.55	XMS-CASPT2/AVTZ	n.d.	0.06	0.42	0.42
MAE	-	-	-	-	-	-
MSE	-	-	-	-	-	-
Count	23.00	-	-	-	-	-

6.6 Distribution of M Diagnostics

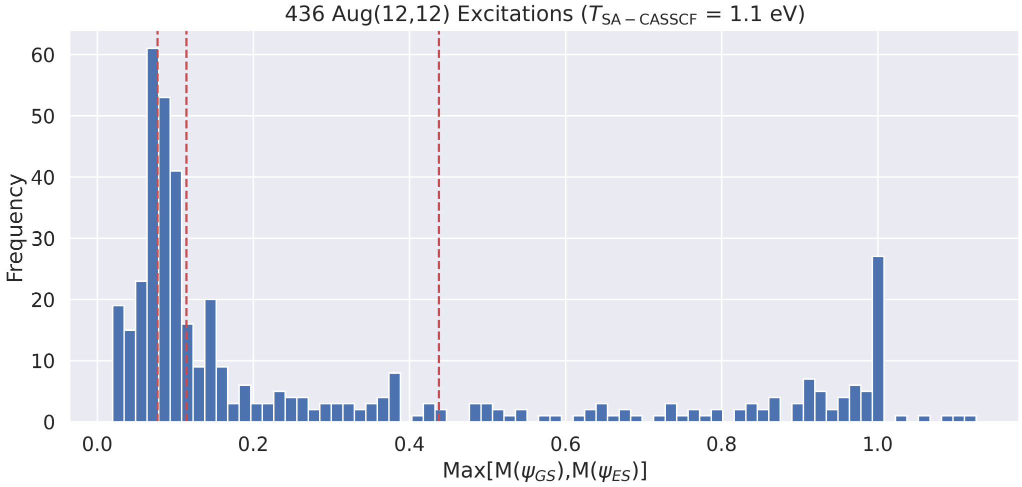

Figure 20: Distribution of calculated maximum M diagnostics Max[M(ψ_{GS}),M(ψ_{ES})] for all 436 excitations included by $T_{\text{SA-CASSCF}}$ = 1.1 eV. Quartile lines are shown at 25% (M=0.08), 50% (M=0.11), and 75% (M=0.44).

6.7 Comparison to Other Methods on Excitations with Low M Diagnostic

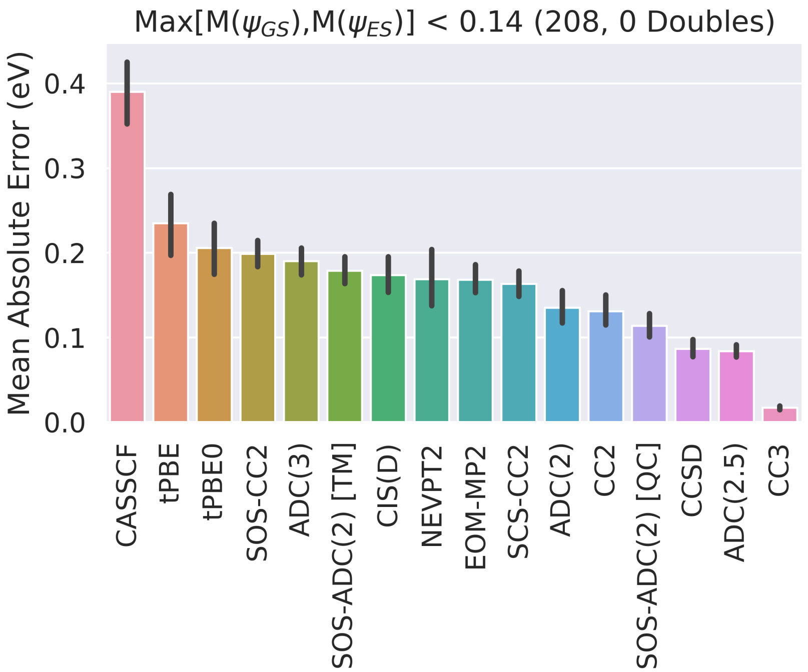

Figure 21: MAE comparison of the 208 Aug(12,12) excitations included by $T_{\text{SA}-\text{CASSCF}} = 1.1$ eV and Max[M(ψ_{GS}),M(ψ_{ES})] < 0.14 with data available for all other methods shown. 95% confidence intervals are shown in black.

7 Alternative Parameterizations of htPBE

In this section, we compare the tuning of the λ mixing parameter in hybrid tPBE on different active spaces and basis sets using $T_{\text{SA-CASSCF}} = 1.1$ eV. Additionally we compare the tuning of λ using $T_{\text{tPBE0}} = 0.55$ eV. The use of the tPBE0 cutoff results in more similarity in the behavior of λ between different active spaces and basis set sizes, providing further evidence for this threshold being better able to determine active space reasonability.

7.1 With 1.1 eV SA-CASSCF error threshold

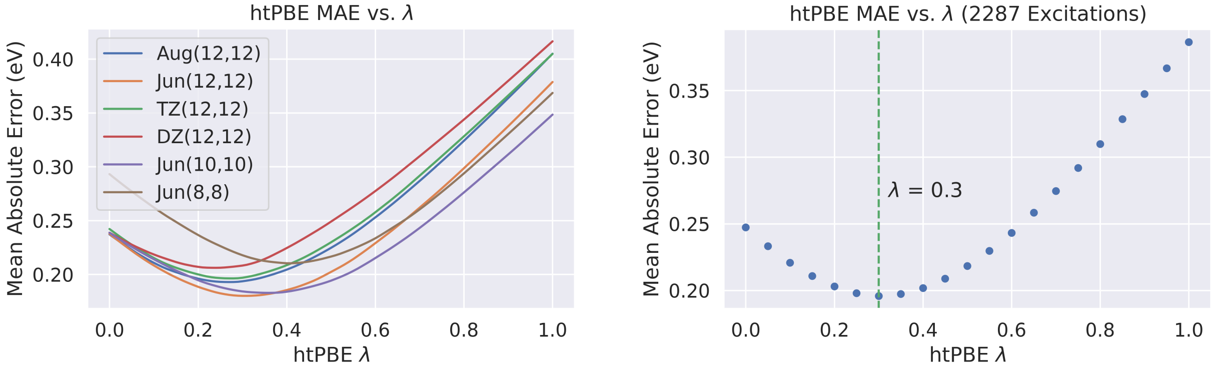

Figure 22: Tuning of the λ parameter in htPBE (defined as $\lambda E_{\text{SA-CASSCF}} + (1-\lambda)E_{\text{tPBE}}$) on various datasets after applying the 1.1 eV SA-CASSCF error threshold. Left: Optimization by active space and basis set. Right: Optimization on all data combined. Most datasets support an optimized value of $\lambda = 0.25$, with the exception Jun(8,8) which appears to support a larger mixing parameter; this is likely due to the increased number of outlier active spaces included by the SA-CASSCF error threshold.

7.2 With 0.55 eV tPBE0 Cutoff

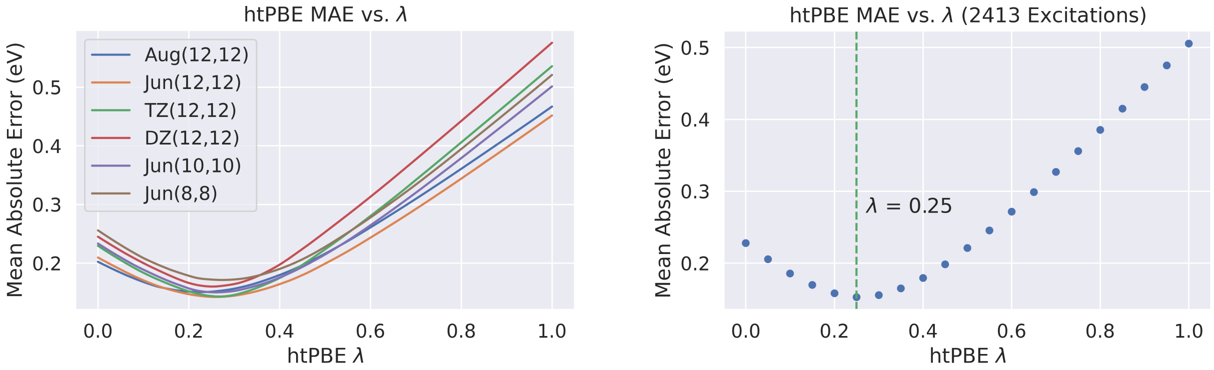

Figure 23: Tuning of the λ parameter in htPBE (defined as $\lambda E_{\text{SA-CASSCF}} + (1-\lambda)E_{\text{tPBE}}$) on various datasets after applying a 0.55 eV tPBE0 error threshold. Left: Optimization by active space and basis set. Right: Optimization on all data combined. Using this more reliable threshold for eliminating poor active spaces, the different active space and basis sets show greater uniformity in their λ preference for 0.25.

8 MC-PDFT Grid Size and Timing

In this section, we compare the timing of tPBE0 calculations by grid size and describe our timing methodology.

8.1 Maximum Deviations from Maximum Grid Fineness

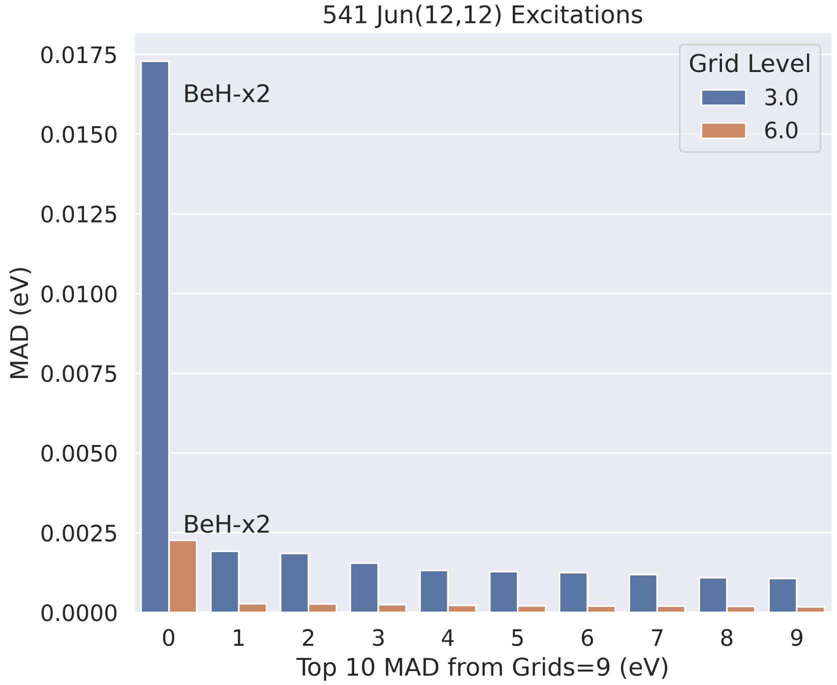

Figure 24: This figure shows the top 10 maximum deviations in computed Jun(12,12) excitation energies between grids_level=3 and grids_level=6 compared to grids_level= 9.

The maximum grid fineness in PySCF is set by the parameter grids_level= 9, with a default of grids_level= 3. The above figure shows that at grids_level= 3, all but one excitation, BeH-x2 ($^2A_1 \rightarrow {}^2B_1$) has a deviation much less than 0.01 eV (it is unknown why this specific excitation is such a stand-out case). We conclude that grids_level= 3 is generally sufficient, and we recommend it for most calculations because grids_level= 9 is found to be about 10× more expensive than the default option (grids_level= 3).

8.2 Timing Methodology

In order to time NEVPT2 and tPBE on equal footing, converged SA-CASSCF wave functions for each excitation were loaded in from disk in separate jobs, and the relevant quantity (the tPBE nonclassical energy or the NEVPT2 perturbative correction) was computed for all states in the

state-averaged manifold. The amount of processors N_{proc} requested for each job was calculated by a function dependent on the number of aug-cc-pVTZ basis functions in the underlying system, N_{aug}:

$$N_{\text{proc}} = \min(2, -51.7 + 0.2484 * N_{\text{aug}}) \tag{15}$$

The amount of memory requested in gigabytes, N_{GB}, was set to $N_{\text{GB}} = 4N_{\text{proc}}$. Equation 15 was informally parameterized based on how the memory use of SA-CASSCF scaled with N_{aug} when memory was more than sufficient. Thus, each excitation was computed with an equivalent amount of resources between different active space sizes and methodologies.

All calculations were conducted on a cluster of Intel Xeon 6248R nodes on the Midway3 cluster hosted by the University of Chicago Research Computing Center. Thus, although the make, model, number of CPUs, and available memory were controlled between all calculations, small hardware differences between specific nodes were not controlled for (although these effects are small enough that we believe our results to be representative).

References

[1] Roothaan, C. C. J. Self-Consistent Field Theory for Open Shells of Electronic Systems. *Rev. Mod. Phys.* **1960**, *32*, 179.

[2] Plakhutin, B.; Gorelik, E.; Breslavskaya, N. Koopmans' Theorem in the ROHF method: Canonical form for the Hartree-Fock Hamiltonian. *J. Chem. Phys.* **2006**, *125*, 204110.

[3] Tsuchimochi, T.; Scuseria, G. E. Communication: ROHF Theory Made Simple. *J. Chem. Phys.* **2010**, *133*, 141102.

[4] Sarkar, R.; Loos, P.-F.; Boggio-Pasqua, M.; Jacquemin, D. Assessing the Performances of CASPT2 and NEVPT2 for Vertical Excitation Energies. *arXiv:2111.15386 [cond-mat, physics:physics]* **2021**, arXiv: 2111.15386.

[5] Véril, M.; Scemama, A.; Caffarel, M.; Lipparini, F.; Boggio-Pasqua, M.; Jacquemin, D.; Loos, P. QUESTDB: A Database of Highly Accurate Excitation Energies for the Electronic Structure Community. *Wiley Interdiscip. Rev. Comput. Mol. Sci.* **2021**, *11*.

[6] Fawcett, T. An Introduction to ROC Analysis. *Pattern Recognit. Lett.* **2006**, *27*, 861.

[7] Youden, W. J. Index for Rating Diagnostic Tests. *Cancer* **1950**, *3*, 32.

[8] Tishchenko, O.; Zheng, J.; Truhlar, D. G. Multireference Model Chemistries for Thermochemical Kinetics. *J. Chem. Theory Comput.* **2008**, *4*, 1208.
